# Leveraging Digital Health Technologies to Assess Older Adults’ Frailty and Nutritional Status: Two Cross-Sectional Studies

**DOI:** 10.2196/77816

**Published:** 2026-04-17

**Authors:** Nunzio Camerlingo, Madisen Wicker, Dimitrios J Psaltos, Nina Shaafi-Kabiri, Hao Zhang, Andrew Messere, Isabelle Messina, Zachary Hayden, Meredith Kelly, Gauri Kamat, Fikret Işık Karahanoğlu, Charmaine Demanuele, Mar Santamaria, David Caouette, Kevin C Thomas

**Affiliations:** 1 Pfizer (United States) Cambridge, MA United States; 2 Chobanian and Avedisian School of Medicine Boston University Boston, MA United States

**Keywords:** digital health technology, older population, frailty, malnutrition, wearable devices

## Abstract

**Background:**

With the rising prevalence of aging worldwide, there is a necessity for reliable and frequent assessments of older individuals’ health status to manage and potentially prevent age-related complications. Digital health technologies (DHTs), such as wearable devices, provide an opportunity to gather objective, continuous, and unobtrusive measurements, enabling effective health management in everyday life.

**Objective:**

We aimed to evaluate the relationship between digital endpoints and frailty, nutritional status, and patient-reported outcomes (PROs) in older individuals, as well as quantified their compliance and comfort with DHTs.

**Methods:**

In two cross-sectional studies, namely the Geriatric Anorexia Studies 1 and 2, including three in-clinic visits and 2-3 weeks of at-home monitoring, 94 participants (mean age 72.98 years, SD 6.28 years) were stratified based on their frailty status (n=39, 41%, nonfrail; n=45, 48%, prefrail; and n=10, 11%, frail) and nutritional status (n=70, 74%, with normal nutrition and n=24, 26%, at risk for malnutrition), as assessed in a clinical setting using the Fried Frailty Score and the Simplified Nutritional Appetite Questionnaire, respectively. We remotely monitored older adults using different DHTs. At home, participants were monitored with a wrist accelerometer for physical activity, continuous glucose monitoring (CGM) for glucose concentration, a digital body scale for weight and body composition, and a digital nutritional scale for meal tracking. Compliance with devices was assessed via wear time and correct at-home usage, while comfort was evaluated using questionnaires. The association between digital endpoints and frailty/nutritional status was investigated via linear regression, followed by ANOVA, and the relationship between digital endpoints collected at home and PROs was evaluated via Spearman’s ρ. Weight and body composition were also assessed during in-clinic visits with a research-grade scale, used to validate the at-home digital body scale measurements, via the intraclass correlation coefficient, Pearson’s R, and Bland-Altman plots with mean bias.

**Results:**

Physical activity digital endpoints collected at home were significantly different across frailty and nutritional groups and significantly correlated with self-reported appetite, fatigue, and physical function (eg, for mean daily activity in the maximum 60 minutes of activity, ρ=0.28, –0.23, and 0.47, respectively). More than 80% of participants reported that all devices were mostly to very acceptable to wear/use: 95% (88/93) for the body scale, 86% (43/50) for CGM, 80% (40/50) for the nutrition scale, and 91% (84/93) for the wrist accelerometer. Compliance ranged from 60% of monitoring days for the digital nutrition scale to 90% for the wrist accelerometer.

**Conclusions:**

Physical activity digital endpoints show clear prognostic significance for frailty and malnutrition in older individuals and reflect self-reported measures. DHTs can be reliably deployed at home for older individuals to measure their physical activity, weight and body composition, glucose concentration, and meal intakes, thus enabling patient-centric, data-centric clinical trials.

**Trial Registration:**

ClinicalTrials.gov NCT04858932; https://clinicaltrials.gov/study/NCT04858932; ClinicalTrials.gov NCT05211973; https://clinicaltrials.gov/study/NCT05211973

## Introduction

Aging is a worldwide phenomenon that is rapidly gaining attention due to the increasing prevalence of older adults and the profound impact it has on individuals and society [1,2]. Aging is associated with a wide variety of comorbidities, such as reduced appetite, undernutrition with associated weight loss and changes in body composition, decreased exercise capacity, and frailty [3]. These conditions are commonly referred to as anorexia of aging (AoA) [4-7].

To diagnose and monitor age-related diseases, typical assessments include, but are not limited to, physical function, weight, and appetite [8]. These assessments are traditionally performed during in-clinic visits using nonscalable devices (eg, research-grade scales to measure weight and body composition) and questionnaires (eg, the Fried Frailty assessment [9]). Notably, in-clinic assessments might not reflect the variability observed in daily life [10,11], because the visits are sporadic and limited in time and space, and participants’ performance can be affected by the presence of observers [12]. In addition, self-reported assessments inherently lack objectivity, particularly for individuals with cognitive impairments, making comparisons between individuals difficult [6]. Finally, in-clinic visits require individuals to travel to clinical trial sites, which can be burdensome, especially for those with reduced mobility and/or those living in rural areas [13].

To overcome the limitations of traditional assessments of age-related diseases and address the need for objective, remote, and unobtrusive high-frequency measurements, the use of digital health technologies (DHTs) has been explored in the literature [11,14]. DHTs refer to a broad category of tools, platforms, and systems that integrate software, hardware, and data analytics to monitor different aspects of health and support clinical decision-making, enhance health care management, and improve patient engagement [15]. This work focuses on DHTs to monitor age-related conditions: wearable accelerometers, digital body weight and nutrition scales, continuous glucose monitoring (CGM) devices, and smartphone apps, whose applications are described next.

Wearable accelerometers, such as smartwatches and lumbar belts, allow for objective measurements of the variability of physical activity and gait in daily life. Their use in older individuals has been widely explored in the literature, showing their potential to unlock new insights into older adults’ frailty and overall health status [16,17].

Digital body scales, deployed in free-living environments, may afford an opportunity to monitor weight and body composition remotely and with more granularity (eg, daily) and to automatically share data with clinical practitioners. This may enable the early detection of weight loss, weight cycling, or sarcopenia, which are associated with disability and mortality in older adults [18,19].

DHTs can also be used to remotely monitor meal intake and glucose concentration, which can indicate alterations in eating behavior, mitigating the risk for malnutrition and potentially preventing the onset of metabolic disorders [20,21]. Handwritten food diaries are often used to report information about meal timings and amounts, but they rely on users’ memory to recall the food consumed, and require the burdensome task of estimating portion size, thus lacking accuracy and reproducibility. In addition, they provide unstructured data, requiring time-consuming and error-prone data harmonization prior to analysis [22-24]. To streamline dietary behavior monitoring, digital nutrition scales can be used to automatically pull meal timings from the network and objectively calculate the serving weight [25,26], resulting in standardized data that can be sent in real time to physicians, dietitians, or nutritionists to accelerate their clinical interpretation. The smartphone apps linked to nutrition scales can provide users with additional features, such as reminders at regular time intervals, which increase compliance and data completeness [27], and the integration of food composition databases, which streamlines the calculation of meals’ nutritional content. More advanced tools, such as the one proposed by Ryu et al [28], can perform automatic estimation of meals’ macronutrient content by leveraging artificial intelligence models to analyze food pictures taken by individuals.

Furthermore, continuous glucose monitoring (CGM) devices represent state-of-the-art systems to track glucose concentration continuously, with wide applications in the diabetes space across different age ranges [29,30]. Although there is evidence for the use of CGM in different disease areas, including obesity [31], eating disorders [32], or following bariatric surgery [33], its application in healthy older individuals has been poorly explored.

The systematic evaluation and early assessment of frailty and malnutrition are crucial to identify individuals at the highest risk for adverse outcomes, allowing the development of individualized care plans, optimization of physical and mental health, and improvement in overall quality of life for the aging population [34]. In this paper, we investigated the use of DHTs to remotely characterize frailty and malnutrition in older adults. For this purpose, we conducted two cross-sectional studies, namely the Geriatric Anorexia Studies 1 and 2 (GAS-1 and GAS-2), in which older individuals, aged 65-96 years, with different frailty and nutritional statuses were monitored both during in-clinic visits and at home with various DHTs: lumbar and wrist accelerometers, a digital body scale, a digital nutrition scale with a smartphone app, and CGM. Our previous study [35] examined the relationship between older individuals’ frailty status and digital endpoints of gait and physical activity using GAS-1 data only. This study integrated GAS-1 and GAS-2 data and broadened the scope by exploring digital endpoints of physical activity, glucose concentration, body composition, and meal composition in relation to (1) the effectiveness of physical activity digital endpoints in distinguishing between frailty and nutritional status and in correlating with self-reported measures and (2) compliance and comfort with DHTs deployed remotely to measure body weight, glucose concentration, and dietary behavior in order to support the design of more patient-centric clinical trials.

## Methods

### Dataset: Geriatric Anorexia Studies

The dataset used in this manuscript combined data collected in two studies: GAS-1 (NCT04858932), which monitored community-dwelling older adults, and GAS-2 (NCT05211973), which monitored a cohort of older individuals in long-term care (LTC) settings, such as nursing homes or assisted-living facilities. Both studies included three in-person visits at the participant’s facility, where traditional assessments were performed, including questionnaires and weight and body composition data collection via research-grade scales, and 2-3 weeks of at-home monitoring, where DHTs were deployed to collect data remotely. Both studies targeted participants aged 65 years or older, with a Montreal Cognitive Assessment (MoCA) score of ≥19. Additional details on the study design and inclusion/exclusion criteria are reported in Multimedia Appendix 1.

### Ethical Considerations

Both studies were reviewed and approved by the Boston University Medical Campus and Boston Medical Center Institutional Review Board (IRB #H-40837 and #H-42048). All participants provided written informed consent prior to enrollment into the study. All study procedures were in accordance with the relevant guidelines/regulations, including data privacy and the Declaration of Helsinki. Data transmission protocols were reviewed by the Boston University Information Technology and the IRB for security and privacy. No identifiable data were used to initialize the devices. Secure File Transfer Protocol (sFTP) was used to transfer data between Boston University and Pfizer, following Good Clinical Practice guidelines, Boston University Information Technology, and Spivack Center for Clinical and Translational Neuroscience policies.

### DHTs Used in the Two Studies

Physical activity data were collected using the GENEActiv accelerometer (Activinsights Ltd), which was worn on the nondominant wrist. A lumbar accelerometer was used only in GAS-1, and the related gait endpoints have been discussed elsewhere [35].

Weight and body composition measurements were collected using two scales, the Tanita MC-780U scale (Tanita Corporation of America) and the Renpho ES-BR003 body scale (Renpho Joicom Corporation). The former, used only during in-person visits by study staff, is a Food and Drug Administration (FDA)–cleared scale that uses bioelectrical impedance analysis (BIA) from footplate and hand electrodes [36] and stores data on a wired computer. The latter, used both during in-clinic visits and at home, is a cost-effective, rechargeable, consumer-grade scale that uses lower extremity BIA and stores data through a connection to its own app downloaded on the iPhone XR (Apple Inc) provided. Participants were instructed to use the Renpho body scale every day in the morning upon waking.

The Freestyle Libre Pro CGM (Abbott Laboratories) was used only in GAS-1 to automatically measure the glucose concentration every 15 minutes.

Finally, GAS-1 participants were instructed to use the Renpho ES-SNG01-W smart nutrition scale (Renpho, Joicom Corporation), a digital food scale, paired via Bluetooth with the Gennec app, to capture meals’ weight and integrate nutritional insights from a food database, helping keep an automatic and comprehensive food diary. As a backup collection system, participants photographed the weight displayed on the scale using the iPhone provided. Participants were required to perform these operations both before and after consuming each meal, including prepackaged meals provided to them by Epicured.

### Assessment of Frailty and Nutritional Status

In this research, the Fried Frailty Score (FFS) was used to evaluate frailty. The FFS involves both questionnaires and objective physical performance tasks (ie, unintentional weight loss, self-reported exhaustion, weakness, slow walking speed, and low physical activity), resulting in a total score of 0-5, which is used to stratify individuals in three frailty groups: nonfrail (FFS=0), prefrail (FFS=1-2), and frail (FFS=3-5) [9]. In some studies [35,37], the FFS has also been used to categorize individuals into two robustness groups, robust (FFS=0, ie, nonfrail), and nonrobust (FFS=1-5, ie, combining prefrail and frail).

In this research, the Mini Nutritional Assessment – Short Form (MNA-SF) and the Simplified Nutritional Appetite Questionnaire (SNAQ) were used to evaluate nutritional status. The MNA-SF includes six questions about food intake, recent weight loss, current mobility, psychological stress or acute disease, neuropsychological problems, and the BMI or calf circumference, resulting in a total score of 0-14, indicating malnutrition (≤7), risk for malnutrition (8-11), and normal nutrition (≥12) [38]. The SNAQ is a four-question tool scored using a 5-point Likert-scale also used to evaluate the nutritional status, resulting in a total score of 4-20, where higher scores indicate better appetite. In particular, scores≤14 have been reported to indicate a significant risk for weight loss of >5% within 6 months [39,40] and to be a marker of malnutrition risk in healthy community-dwelling older adults [41].

### Patient-Reported Outcomes

Patient-reported outcomes (PROs) were measured during in-clinic visits and at home on paper by participants. Appetite and fatigue PROs were measured daily. The question on appetite was “How would you rate your appetite during the past 24 hours from 0 to 10 (0=no appetite, 10=very good appetite)?,” while the fatigue question was “How would you rate your physical fatigue during the past 24 hours from 0 to 10 (0=no fatigue, 10=worst-possible fatigue)?” These questions were developed by Pfizer based on concept elicitation interviews and cognitive debriefing of questions and response options with the population of interest, following the “FDA Patient-Focused Drug Development Guidance Series for Enhancing the Incorporation of the Patient’s Voice in Medical Product Development and Regulatory Decision Making” [42]. Finally, the well-known PROMIS (Patient-Reported Outcomes Measurement Information System) Physical Function 10a was used during the in-clinic visits [43].

Comfort and wearability questionnaires were used at the last visit to gather feedback on the use of DHTs. The questionnaires included 10 items, with rated questions and free-text comments, reflecting what has been already used in previous studies across different populations [44,45]. In this research, the scores of the questions were not aggregated, but we focused only on the following two questions provided separately per device: “Please rate the ease of wearing and/or using the device,” where participants could answer on a 1-7 scale (1=very acceptable, 7=very unacceptable), and “Would you be willing to use the device in the future, and if so, for how long?,” where participants could select from “No,” “Yes, less than 1 week,” “Yes, 1-4 weeks,” and “Yes, more than 4 weeks”.

### Endpoint Generation

Physical activity endpoints were derived from the GENEActiv wrist accelerometer using the vendor-supplied proprietary algorithm. This algorithm aggregates the 50 Hz triaxial raw accelerometry data into 1-minute summaries by extracting the signal vector magnitude (SVMg). Based on SVMg thresholds, the algorithm distinguishes wear from nonwear periods, and during wear periods, it classifies activity as sleep, sedentary, light, moderate, and vigorous. Visit days were excluded from the analysis, as they contained only partial recordings. In addition, only participants with at least 4 compliant days were included in the analysis. For each compliant day, the following physical activity endpoints were derived: time in sedentary activity (hours), time in nonsedentary activity (hours), time in sedentary-to-light physical activity (SLPA; hours), time in moderate-to-vigorous physical activity (MVPA; hours), mean SVMg (arbitrary units [a.u.]), 95th percentile of SVMg (a.u.), mean SVMg during the maximum 6 minutes of activity (M6min; a.u.), and mean SVMg during the maximum 60 minutes of activity (M60min; a.u.). The last two endpoints have been used in the literature to quantify the average activity during the most active 6 and 60 minutes of the day and have shown significant association with traditional measures of functional capacity in older individuals [46]. Finally, physical activity endpoints were averaged across compliant days for each participant.

The following measurements were collected using the Renpho body scale and the Tanita scale: weight (kg), muscle mass (kg), fat mass (kg), fat-free mass (kg), bone mass (kg), and water mass (kg). All body composition measurements were converted to percentages of weight. In-clinic and at-home body measurements were averaged across the three visits and at-home monitoring days per participant, respectively.

To obtain meal-related endpoints, timestamps, which were manually transcribed from each meal photo by two scientists and an independent reviewer, were associated with a meal weight. Weight entries from Gennec and the photos were then paired using their timestamps and meal names. In some cases, weight data were available only from Gennec, if participants did not take the photo correctly (eg, it was too blurry, or the weight was not photographed) or if participants did not take a photo at all. In other cases, weight data were available only from the photo if participants did not connect Gennec to the Renpho nutrition scale, they did not open the app to trigger a new meal recording, or the meal was not left long enough on the scale for the app to record the weight. When the weight was available from both sources, the average weight was computed for both pre- and postmeal entries. The meal net weight (g) was computed as the difference between the post- and premeal weights.

In the case of prepackaged meals, the meal macronutrient content was available, including energy (kcal), fat (g), sodium (g), potassium (g), carbohydrates (g), protein (g), and potassium (g). The net macronutrient content of the meal was computed by applying the percentage of consumed meal weight (ratio of meal net weight and premeal weight) to the macronutrient content of the prepackaged meal, under the assumption that participants consumed the same percentage of macronutrients in the meal. The net macronutrient content was computed only for the prepackaged meals. Meal duration was obtained as the time that elapsed between the pre- and postmeal times. The averages of net weight, energy, macronutrient content, and duration across meals were extracted per participant.

The following endpoints were computed from the Abbott Freestyle Libre Pro CGM data for each at-home monitoring day: mean (SD; mg/dL), minimum (mg/dL), maximum (mg/dL), time in range (TIR, %, defined as the percentage time within 70-180 mg/dL), time below range (TBR, %, defined as the percentage time below 70 mg/dL), and time above range (TAR, %, defined as the percentage time above 180 mg/dL). CGM endpoints were averaged across monitoring days per participant.

PROMIS Physical Function 10a scores were converted to *t*-scores (mean 50, SD 10, representing the US general population) according to standard guidelines [43]. PROMIS Physical Function 10a *t*-scores as well as appetite and fatigue PRO scores were averaged across monitoring days per participant.

### Statistical Analysis

The relationship between frailty and nutritional groups was investigated via chi-square tests performed between pairs of categories (ie, FFS vs MNA-SF, FFS vs SNAQ, MNA-SF vs SNAQ), with scatter plots and Spearman’s correlation.

The endpoints derived from DHTs at home were tested to assess whether they distinguished the frailty or nutritional groups. For this purpose, a linear regression model was leveraged, with the average endpoint as a dependent variable and the group (ie, nonfrail/prefrail/frail, nonrobust/robust, or normal nutrition/at risk for malnutrition), age, and sex as independent variables. Height was also added as an independent variable for models investigating differences in weight and body composition endpoints.

Model residuals were characterized in terms of normality via the Shapiro-Wilk test and homogeneity of the variance across groups via Levene’s test. The presence of an overall statistically significant association between endpoints and groups was investigated via one-way ANOVA in the case of normality and homogeneity of the residuals, Welch’s ANOVA in the case of normality but nonhomogeneity of the residuals, or the Kruskal-Wallis test in the case of nonnormality of the residuals. For *P*<0.1, post hoc pairwise tests were performed to assess differences between pairs of frailty groups via least square comparisons following ANOVA or Wilcoxon tests following the Kruskal-Wallis test. The 0.1 threshold was chosen to flag digital endpoints exhibiting potential trends across groups, thereby reducing type II error. No correction for multiple comparisons was applied [47].

The association between digital endpoints with PRO scores was assessed using Spearman’s correlation (ρ) to account for outliers.

Agreement between the Renpho body scale (test device) and the Tanita scale (reference device) endpoints collected during in-clinic visits was assessed using the intraclass correlation coefficient (ICC; two-way random-effects model, absolute agreement) following benchmarks reported by Cicchetti et al [48]: ICC≤0.4, poor agreement; ICC=0.4-0.59, fair agreement; ICC=0.6-0.74, good agreement; and ICC=0.75-1, excellent agreement. The mean difference (bias), limits of agreement (LoA), mean absolute difference, mean percentage error, and Pearson’s correlation coefficient (R) were also computed. Scatter plots and Bland-Altman plots with 95% LoA were used to visualize agreement.

All statistical analyses were performed in R version 3.4.2 (R Foundation for Statistical Computing) using the following main packages: *car* for type III ANOVA, *BlandAltmanLeh* for Bland-Altman plots, and *psych* for the ICC.

## Results

### Demographics

In total, 94 older individuals (n=48, 51%, females) aged 65-96 (mean 72.98, SD 6.28) years with a BMI of 17.3-50.4 (mean 25.81, SD 4.60) kg/m^2^ were recruited across GAS-1 (n=50, 53%) and GAS-2 (n=44, 47%) and were monitored with DHTs at home for 8-16 (mean 12.43, SD 1.20) days (excluding visit days). Of the 94 participants, 7 (7%) reported partial use of walking aids (eg, cane, walker, or leg braces), and 6 (6%) reported previous hip or knee replacement.

According to the MNA-SF, nutritional status was classified as “normal” for 67 (71%) participants (mean age 72.39, SD 5.57 years), of which 28 (42%) were females, and “at risk for malnutrition/malnourished” for 27 (29%) participants (mean age 74.44, SD 7.69 years), of which 20 (74%) were females. According to the SNAQ, nutritional status was classified as “normal” for 70 (74%) participants (mean age 73.58, SD 6.17 years), of which 36 (51%) were females, and “at risk for malnutrition/weight loss” for 24 (26%) participants (mean age 71.21, SD 6.39 years), of which 12 (50%) were females. Participants’ characteristics are presented in Table 1, stratified by frailty phenotype. Chi-square tests showed expected significant associations between groups (FFS vs MNA-SF: *P*<.001; FFS vs SNAQ: *P*<.01; MNA-SF vs SNAQ: *P*<.05). Scatter plots with Spearman’s correlation between the FFS, MNA-SF, and SNAQ are reported in Figure S1 in Multimedia Appendix 1.

**Table 1:** Participants’ demographics characteristics and compliance with digital tools, stratified by frailty group.^a^

Characteristics		Nonfrail group (n=39; FFS^b^=0)	Prefrail group (n=45; FFS=1-2)	Frail group (n=10; FFS≥3)	Total participants (N=94)
Enrolled female participants, n (%)		20 (51)	18 (40)	10 (100)	48 (51)
Age (years), mean (SD, range)		70.79 (3.34, 65.00-81.00	74.60 (6.62, 65.00-93.00	74.20 (10.53, 65.00-96.00	72.98 (6.28, 65.00-96.00
BMI (kg/m^2^), mean (SD, range)		25.06 (3.39, 18.60-29.80	26.52 (3.51, 20.80-35.30	25.49 (10.23, 17.30-50.40	25.81 (4.60, 17.30-50.40
**Race** **, n (%)**					
	Black/African American	5 (13)	11 (24)	2 (20)	18 (19)
	White	33 (85)	33 (73)	7 (70)	73 (78)
	More than one race	1 (2)	1 (3)	1 (10)	3 (3)
**FFS** **, n (%)**					
	0	39 (100)	—^c^	—	39 (41)
	1	—	33 (73)	—	33 (35)
	2	—	12 (27)	—	12 (13)
	3	—	—	7 (70)	7 (7.4)
	4	—	—	3 (30%)	3 (3.2%)
**MNA-SF^d^** **score**					
	Normal (score 12-14), n (%)	31 (79)	35 (78)	1 (10)	67 (71)
	At risk for malnutrition/malnourished (score 0-11), n (%)	8 (21)	10 (22)	9 (90)	27 (29)
	Total (score 0-14), median (IQR, range)	13.00 (2.00, 10.00-14.00)	13.00 (2.00, 8.00-14.00)	9.50 (4.5, 3.00-14.000)	13.00 (3.00, 3.00-14.00)
**SNAQ^e^** **score**					
	Normal (score 15-20), n (%)	31 (79)	36 (80)	3 (30)	70 (74)
	At risk for weight loss/malnutrition (score 4-14), n (%)	8 (21)	9 (20)	7 (70)	24 (26)
	Total (score 4-20), median (IQR, range)	16.00 (2.00, 12.00-18.00)	15.00 (2.00, 11.00-18.00)	12.00 (5.50, 6.00-16.00)	15.50 (2.75, 6.00-18.00)
At-home monitoring days (excluding visit days), mean (SD, range)		12.30 (0.67, 11.00-13.00)	12.44 (1.98, 8.00-16.00)	12.44 (1.31, 9.00-15.00)	12.43 (1.20, 8.00-16.00)
Compliance with wrist accelerometer (%), median (IQR, range)		100 (3.50, 42.00-100.00)	100 (9.00, 21.00-100.00)	100 (8.00, 54.00-100.00)	100 (8.00, 21.00-100.00)
Compliance with Renpho body scale (%), median (IQR, range)		92.86 (8.01, 40.00-100.00)	92.30 (9.09, 0.00-100.00)	91.99 (38.78, 45.45-100.00)	92.30 (8.33, 0.00-100.00)
Compliance with CGM^f^ (%), median (IQR, range)		100 (11.54, 8.33-100.00)	100 (44.23, 25.00-100.00)	100 (0.00, 75.00-100.00)	100 (24.04, 8.33-100.00)
Compliance with Renpho nutrition scale (%), median (IQR, range)		81.82 (45.64, 0.00-100.00)	78.57 (50.00, 0.00-100.00)	7.14 (16.07, 0.00-71.43)	78.57 (76.23, 0.00-100.00)

^a^Continuous data are reported as the mean (SD, range), categorical data are reported as the median (IQR) or n (%), and compliance data are reported as the median (IQR, minimum-maximum).

^b^FFS: Fried Frailty Score.

^c^Not applicable.

^d^MNA-SF: Mini Nutrition Assessment – Short Form.

^e^SNAQ: Simplified Nutritional Appetite Questionnaire.

^f^CGM: continuous glucose monitoring.

### Physical Activity at Home Distinguishes Frailty and Nutritional Status and Correlates With PROs

**Figure 1:**
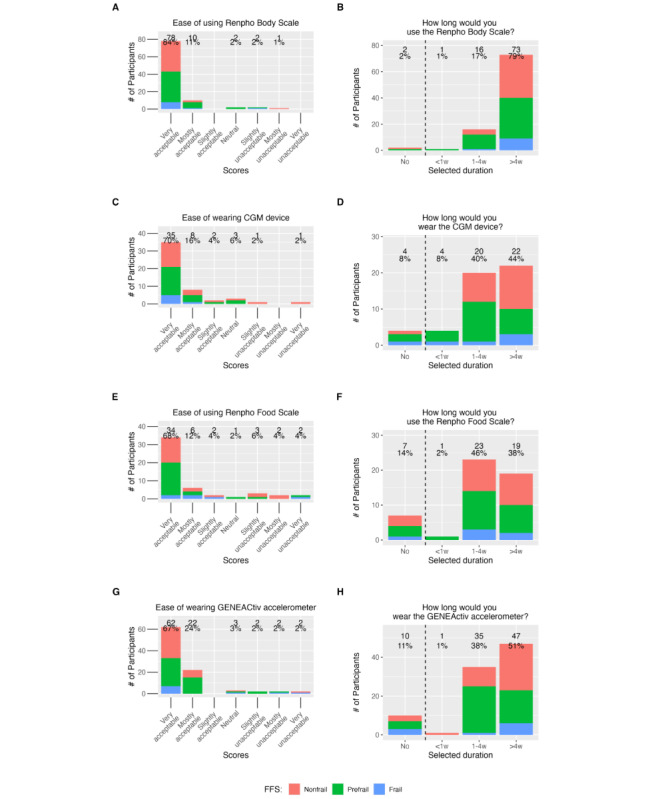
Scores from the comfort and wearability questionnaires, stratified across frailty groups. (A, B) Renpho Body Scale, (C, D), CGM, (E, F), Renpho Food Scale, and (G, H) GENEActiv accelerometer. The color indicates the frailty phenotypes: nonfrail (red), prefrail (green), and frail (blue). Most of the participants found the devices easy to wear or use and indicated their willingness to wear or use them for at least 1 week regardless of their frailty status/score. CGM: continuous glucose monitoring; FFS: Fried Frailty Score.

A compliant day was defined as a day with at least 18 hours of wear time, in agreement with previous literature [49,50], resulting in a mean percentage compliance of 92.04% (SD 17.32%, range 21.00%-100.00%) across participants (Table 1). Data from 3 (3%) participants with less than 4 compliant days were excluded from the analysis, resulting in 91 (97%) compliant participants. During compliant days, participants had a mean wear time of 23.13 (SD 0.82, range 19.91-24.00) hours/day. Questionnaire responses showed that of 93 participants, 84 (91%) found the wrist accelerometer mostly to very acceptable to wear (Figure 1A), and 82 (89%) indicated that they would wear the wrist accelerometer for more than 1 week (Figure 1B).

**Figure 2:**
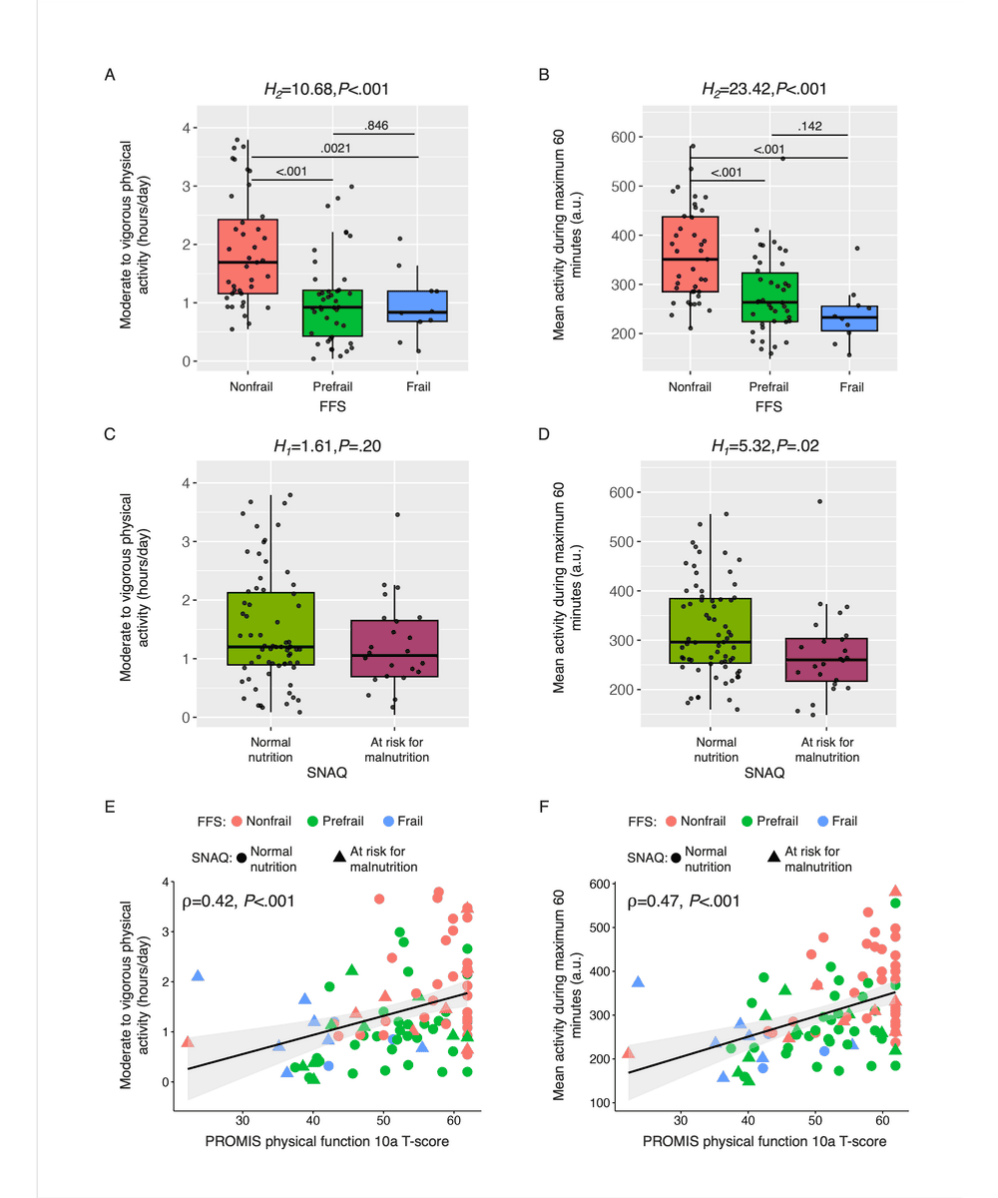
(A, C, E) MVPA and (B, D, F) and M60min across (A, B) frailty groups, (C, D) across nutritional groups, and (E, F) against PROMIS Physical Function 10a t-scores. (A-D) Data are reported in boxplot representation: central horizontal lines represent the median, boxes mark the IQR, black lines are the whiskers, and black dots indicate outliers. H-scores, degrees of freedom, and *P* value of Kruskal-Wallis checking the association between average endpoints and groups are reported in the title. Colors indicate the frailty group (nonfrail [red], prefrail [green], and frail [blue]) or the nutritional group (normal nutrition [green] and at risk for malnutrition [purple]). Frail participants showed significantly lower MVPA and M60min, and participants at risk for malnutrition showed significantly lower M60min and a smaller MVPA median value. (E, F) Data are reported as scatter plots, where each dot represents the average across participants’ daily measurements. Colors indicate the frailty group, while shapes indicate the nutritional group. Spearman’s correlation and related *P* values are annotated. Significant correlation with self-reported physical function was observed for both MVPA and M60min. FFS: Fried Frailty Score; M60min: maximum 60 minutes of activity; MVPA: moderate-to-vigorous physical activity; PROMIS: Patient-Reported Outcomes Measurement Information System; SNAQ: Simplified Nutritional Appetite Questionnaire.

ANOVA showed a significant effect of frailty status on all physical activity endpoints except SLPA (Table 2). Time spent in sedentary activity, nonsedentary activity, and MVPA (Figure 2A) was significantly different across frailty groups: the nonfrail group spent significantly more time in nonsedentary activity compared to both prefrail and frail groups, significantly more time in MVPA compared to both prefrail and frail groups, and significantly less time in sedentary compared to the frail group. In addition, the mean and 95th percentile of SVMg, as well as M6min and M60min (Figure 2B), were significantly different across frailty groups. The results were concordant when comparisons were performed across the two robustness groups, with the nonrobust group showing significantly higher time in sedentary activity and lower time in nonsedentary activity, MVPA, mean SVMg, 95th percentile of SVMg, M6min, and M60min, compared to the robust group.

Furthermore, a significant age effect was observed for time spent in nonsedentary activity (*P*<.01), MVPA (*P*<.01), mean SVMg (*P*<.05), and 95th percentile of SVMg (*P*<.01), while a significant effect of sex was observed for M6min (*P*<.001), with female participants showing a lower M6min (mean 528, SD 135 a.u.) compared to male participants (mean 679.2, SD 270 a.u.).

Physical activity endpoints related to high-intensity activity, that is, M6min and M60min (Figure 2D) were significantly different across SNAQ groups. None of the physical activity endpoints showed a significant difference across MNA-SF groups (Table 3).

Two participants did not complete the appetite and fatigue questionnaires at home, resulting in a total of 92 (98%) respondents. The question on appetite was answered at home for an average of 13.31 (SD 2.28) days per participant, with an average score of 8.02 (SD 1.87) across participants. The average appetite score differed across frailty groups (*F*_2,87_=4.53, *P*=.01), robustness (*F*_1,88_=5.24, *P*=.02), and nutritional (MNA-SF: *F*_1,88_=4.76, *P*=.03; SNAQ: *F*_1,88_=18.42, *P*<.001) groups, as illustrated in Figure S5 in Multimedia Appendix 1. M6min (ρ=0.3, *P*<.01) and M60min (R=0.28, *P*<.01) showed a significant correlation with the average appetite score.

The question on fatigue was answered at home for an average of 13.27 (SD 2.34) days per participant, with an average score of 2.95 (SD 2.09) across participants. The average fatigue score differed across frailty groups (*F*_2,87_=10.01, *P*<.001), robustness (*F*_1,88_=14.52, *P*<.001), and nutritional (MNA-SF: *F*_1,88_=4.01, *P*<.05; SNAQ: *F*_1,88_=6.01, *P*<0.5) groups, as illustrated in Figure S6 in Multimedia Appendix 1. M60min (ρ=–0.23, *P*<.05) showed a significant but weak correlation with the average fatigue score.

All participants completed the PROMIS Physical Function 10a questionnaire, resulting in a mean *t*-score of 52.28 (SD 9.01) across participants. The average *t*-score differed across frailty (*F*_2,89_=12.68, *P*<.001), robustness (*F*_1,90_=11.6, *P*<.001), and nutritional (MNA-SF: *F*_1,90_=4.83, *P*<.05; SNAQ: *F*_1,90_=20.2, *P*<.001) groups, as illustrated in Figure S7 in Multimedia Appendix 1. The time spent in nonsedentary activity (ρ=0.35, *P*<.001), the time spent in MVPA (ρ=0.42, *P*<.001; Figure 2E), the mean SVMg (ρ=0.32, *P*<.01), the 95th percentile of SVMg (ρ=0.4, *P*<.001), M6min (ρ=0.34, *P*<.01), and M60min (ρ=0.47, *P*<.001; Figure 2F) were significantly correlated with the PROMIS Physical Function 10a *t*-scores.

**Table 2:** Physical activity endpoints averaged across all participants and across frailty and robustness groups.

Physical activity endpoint	Total participants (N=91), mean (SD); ANOVA *F*-score, *P* value/Kruskal-Wallis *H* value, *P* value^a^	Nonfrail/robust group (n=39), mean (SD); *P* value vs frail group	Prefrail group (n=42), mean (SD); *P* value vs nonfrail group	Frail group (n=10), mean (SD); *P* value vs prefrail group	Nonrobust group (n=52), mean (SD); ANOVA *F*-score, *P* value/Kruskal-Wallis *H* value, *P* value^a^
Time spent in sedentary activity (hours/day)^b^	11.68 (2.00); *F*_2,86_=3.50, *P*=.04	11.03 (2.11); .02	12.03 (1.82)*;* .12	12.77 (1.57)*;* .15	12.17 (1.78); *F*_1,87_=4.78, *P*=.03
Time spent in nonsedentary activity (hours/day)^b^	2.92 (1.48); *H*_2_=13.04, *P*=.002	3.59 (1.38); .02	2.45 (1.41); <.001	2.33 (1.18); .90	2.43 (1.36); *H*_1_=12.97, *P*<.001
Time spent in SLPA^c^ (hours/day)	13.20 (1.94); *F*_2,86_=2.14, *P*=.12	12.72 (2.07); —^d^	13.43 (1.74); —	14.13 (1.86); —	13.56 (1.76); *F*_1,87_=2.86, *P*=.10
Time spent in MVPA^e^ (hours/day)^b^	1.41 (0.93); *H*_2_=20.68, *P*<.001	1.90 (0.96); .002	1.05 (0.75); <.001	0.97 (0.58); .85	1.04 (0.72); *H*_1_=20.60, *P*<.001
SVMg^f^ (a.u.^g^)^b^	119.9 (29.0); *H*_2_=9.05, *P*=.01	130.3 (27.6)*;* .04	113.0 (26.5)*;* .01	108.1 (23.6)*;* .61	112.1 (25.9); *H*_1_=8.81, *P*=.003
95th Percentile SVMg (a.u.)^b^	348.6 (86.2); *F*_2,86_=8.19, *P*<.001	394.2 (79.4); .01	316.6 (77.5); <.001	305.3 (66.6); .81	314.4 (75.0); *F*_1,87_=16.50, *P*<.001
M6min^h^ (a.u.)^b^	602.8 (225.0); *H*_2_=16.13, *P*<.001	705.9 (242.0); .002	636.0 (189.0); <.001	480.7 (110.0); .50	525.4 (177.0); *H*_1_=15.57, *P*<.001
M60min^i^ (a.u.)^b^	307.8 (96.6); *H*_2_=23.42, *P*<.001	360.2 (93.7); <.001	275.9 (81.9); <.001	238.1 (60.1); .14	268.6 (79.1); *H*_1_=21.34, *P*<.001

^a^*F*-scores and *P* values for ANOVA or *H* values and *P* values for Kruskal-Wallis tests for the effect of frailty and robustness status on physical activity endpoints are reported, along with *P* values of post hoc pairwise comparisons.

^b^Physical activity endpoints significantly different across both frailty and robustness groups.

^c^SLPA: sedentary-to-light physical activity.

^d^Not computed.

^e^MVPA: moderate-to-vigorous physical activity.

^f^SVMg: signal vector magnitude.

^g^a.u.: arbitrary units.

^h^M6min: maximum 6 minutes of activity.

^i^M60min: maximum 60 minutes of activity.

**Table 3:** Physical activity endpoints averaged across nutritional groups, as assessed using the MNA-SF^a^ and the SNAQ^b^.

Physical activity endpoint	MNA-SF		SNAQ	
	Normal nutrition (n=65), mean (SD); ANOVA *F*-score, *P* value/Kruskal-Wallis *H* value, *P* value^c^	At risk for malnutrition (n=26), mean (SD); ANOVA *F*-score, *P* value/Kruskal-Wallis *H* value, *P* value^c^	Normal nutrition (n=67), mean (SD); ANOVA *F*-score, *P* value/Kruskal-Wallis *H* value, *P* value^c^	At risk for malnutrition (n=24), mean (SD); ANOVA *F*-score, *P* value/Kruskal-Wallis *H* value, *P* value^c^
Time spent in sedentary activity (hours/day)	11.76 (2.01); *F*_1,87_=0.60, *P*=.44	11.47 (2.00); *F*_1,87_=0.60, *P*=.44	11.78 (1.95); *F*_1,87_=0.18, *P*=.67	11.41 (2.16); *F*_1,87_=0.18, *P*=.67
Time spent in nonsedentary activity (hours/day)	2.99 (1.48); *H*_1_=0.31, *P*=.58	2.75 (1.49); *H*_1_=0.31, *P*=.58	3.01 (1.51); *H*_1_=0.572, *P*=.45	2.70 (1.39); *H*_1_=0.572, *P*=.45
Time spent in SLPA^d^ (hours/day)	13.31 (1.91); *F*_1,87_=1.16, *P*=.29	12.92 (2.02); *F*_1,87_=1.16, *P*=.29	13.30 (1.79); *F*_1,87_=0.36, *P*=.55	12.92 (2.32); *F*_1,87_=0.36, *P*=.55
Time spent in MVPA^e^ (hours/day)	1.44 (0.95); *H*_1_=0.37, *P*=.54	1.31 (0.89); *H*_1_=0.37, *P*=.54	1.48 (0.97); *H*_1_=1.61, *P*=.20	1.19 (0.78); *H*_1_=1.61, *P*=.20
SVMg^f^ (a.u.^g^)	120.8 (29.3); *H*_1_=0.367, *P*=.54	117.6 (24.7); *H*_1_=0.367, *P*=.54	121.0 (28.5); *H*_1_=0.342, *P*=.56	116.7 (26.7); *H*_1_=0.342, *P*=.56
95th Percentile SVMg (a.u.)	353.2 (87.0); *F*_1,87_=0.08, *P*=.78	337.0 (84.5); *F*_1,87_=0.08, *P*=.78	354.4 (86.8); *F*_1,87_=3.32, *P*=.07	332.3 (83.9); *F*_1,87_=3.32, *P*=.07
M6min^h^ (a.u.)	628.8 (248.0); *H*_1_=1.21, *P*=.27	537.6 (136.7); *H*_1_=1.21, *P*=.27	637.3 (236.0); *H*_1_=7.16, *P*=.007	506.3 (158.0); *H*_1_=7.16, *P*=.007
M60min^i^ (a.u.)	316.5 (98.7); *H*_1_=1.30, *P*=.25	286.2 (89.3); *H*_1_=1.30, *P*=.25	320.5 (96.1); *H*_1_=5.32, *P*=.02	272.4 (90.5); *H*_1_=5.32, *P*=.02

^a^MNA-SF: Mini Nutrition Assessment – Short Form.

^b^SNAQ: Simplified Nutritional Appetite Questionnaire.

^c^*F*-scores and *P* values for ANOVA or *H* values and *P* values for Kruskal-Wallis tests for the effect of nutritional status on physical activity endpoints are reported.

^d^SLPA: sedentary-to-light physical activity.

^e^MVPA: moderate-to-vigorous physical activity.

^f^SVMg: signal vector magnitude.

^g^a.u.: arbitrary units.

^h^M6min: maximum 6 minutes of activity.

^i^M60min: maximum 60 minutes of activity.

### DHTs Can Enable Patient-Centric Clinical Trials in Older Individuals

The accuracy of the weight and body composition measurements from the Renpho Body Scale and the Tanita scale was compared during in-clinic visits. Perfect agreement (ICC=1) and correlation (R=1) were observed for weight. Mean percentage errors<1% were found for weight and water mass and <4% for muscle mass, fat mass, and fat-free mass. Interestingly, a higher error and nonhomogeneous bias were observed when considering absolute rather than percentage measurements. A comprehensive comparison between the Renpho Body Scale and the Tanita scale is reported in Multimedia Appendix 1.

Compliant days at home using the Renpho Body Scale were defined as those with at least one measurement, resulting in an average percentage compliance of 88.36% (SD 20.49%) across participants. Only 1 (1%) participant did not collect any at-home measurements (0% compliance; this participant felt unsafe using the scale without assistance), while 39 (41%) participants weighed themselves every day (100% compliance). Furthermore, 2 (2%) participants were unable to connect the body scale to the app; thus, only their weight was captured, not the body composition measures.

Questionnaire responses showed that 88 (95%) of 93 participants found the Renpho Body Scale mostly to very acceptable to use, and 89 (97%) of 92 participants indicated that they would use the body scale for more than 1 week.

ANOVA did not show any statistically significant difference across frailty, robustness, or nutritional groups for any of the Renpho Body Scale endpoints. As expected, height showed a significant effect on weight (*P*<.001). In addition, sex showed a significant effect on muscle mass (females: mean 64.96%, SD 8.37%; males: mean 72.85%, SD 4.90%; *P*<.001), fat mass (females: mean 30.92%, SD 8.87%; males: mean 23.30%, SD 5.13%; *P*<.01), water mass (females: mean 47.46%, SD 6.21%; males: mean 55.32%, SD 3.84%; *P*<.001), and fat-free mass (females: mean 69.08%, SD 8.88%; males: mean 76.70%, SD 5.13%; *P*<.01).

Muscle mass (ρ=–0.32, *P*<.01), bone mass (ρ=–0.23, *P*<.05), fat mass (ρ=0.32, *P*<.01), fat-free mass (ρ=–0.32, *P*<.01), and water mass (ρ=–0.31, *P*<.01) showed a significant but weak correlation with self-reported fatigue. Similarly, muscle mass (ρ=0.32, *P*<.01), bone mass (ρ=0.21, *P*<0.05), fat mass (ρ=–0.32, *P*<.01), fat-free mass (ρ=0.32, *P*<.01), and water mass (ρ=0.31, *P*<.01) showed significant correlations with self-reported physical function.

Of 50 participants, CGM data were available for 43 (86%) participants: For 2 (4%) participants, the devices failed to connect to the reader at the last visit; 1 (2%) participant lost the device during the study; for 3 (6%) participants, the device fell off during the second day of monitoring; and for 1 (2%) participant, the device appeared damaged at the last visit (with the adhesive partially removed and the needle bent sideways).

Excluding visit days, CGM data were recorded for an average of 10.12 (SD 3.37, range 1.00-14.00) days, corresponding to an average of 84.27% (SD 26.68%, range 8.33%-100.00%) of the monitoring period. In total, 25 (50%) participants wore the CGM every day (100% compliance). During days with CGM recordings, the average wear time was 23.23 (SD 1.39, range 18.00-24.00) hours across participants.

Questionnaire responses showed that 43 (86%) of 50 participants found the CGM device mostly to very acceptable to wear, and 42 (84%) of 50 participants indicated that they would use the CGM for more than 1 week.

ANOVA did not show any statistically significant differences across frailty, robustness, or nutritional groups for any CGM endpoints. However, the frail group showed a higher TBR (~7%), and a lower TIR (~8%) compared to nonfrail and prefrail groups (Table S3 in Multimedia Appendix 1).

The minimum glucose concentration showed a significant correlation with self-reported appetite (ρ=0.32, *P*=.04) and self-reported fatigue (ρ=–0.31, *P*=.05).

Participants were considered compliant with using the Renpho Nutrition Scale only if they weighed a meal both before and after consumption. The percentage of days with at least one compliant meal entry was an average of 60.29% (SD 37.00%, range 0.00%-100.00%) across all participants. In addition, 1 (2%) participant was unable to record any meals with the nutrition scale throughout the study, while 4 (8%) participants weighed their meals only once (either before or after consumption) throughout the study. Furthermore, 5 (10%) participants were compliant every day (100% compliance), and 34 (68%) reported at least three meals on an average of 53.40% (SD 24.68%, range 6.66%-92.31%) of the monitoring days. Finally, across participants, a mean total of 25.34 (SD 23.24, range 0.00-121.00) meals were correctly entered during the study using the Renpho Nutrition Scale, resulting in 1267 total meals.

Questionnaire responses showed that 40 (80%) of 50 participants found the nutrition scale mostly to very acceptable to use, and 42 (84%) of 50 participants indicated that they would use the nutrition scale for more than 1 week.

The histograms of meal net weight, meal duration, time between consecutive daily meals, and number of daily meals are reported in Figure S8 in Multimedia Appendix 1. ANOVA showed significant differences between frailty groups only for the net sodium (*F*_2,45_=4.4, *P*=.02) and net potassium (*F*_2,45_=3.6, *P*=.03) content, which were lower in the frail group compared to the nonfrail (*P*<.05) and prefrail (*P*<.05) groups. However, no significant differences across robustness groups were observed for any meal endpoints (Table S3 in Multimedia Appendix 1).

Sex showed significant effects on meal net weight (females: mean 263.3, SD 79.8; males: mean 322.2, SD 72.2; *P*<.05) and meal net protein (females: mean 19.71, SD 6.91; males: mean 25.39, SD 5.14; *P*<.05). Age showed a significant effect on meal duration (*F*_1,45_=9.79, *P*<.01), with older participants being associated with a longer meal duration. When grouping by SNAQ (but not MNA-SF), participants at risk for malnutrition reported a significantly lower number of daily meals (*F*_1,46_=6.89, *P*<.05) and a significantly lower meal net weight (*F*_1,46_=6.83, *P*<.05), as summarized in Table S4 in Multimedia Appendix 1. After adding the meal net weight as a model covariate, participants at risk for malnutrition exhibited a significantly longer meal duration (*F*_1,45_=6.29, *P*<.05).

The meal net weight showed a significant correlation with self-reported appetite (ρ=0.30, *P*<.05) and physical function (ρ=0.48, *P*<.001).

## Discussion

### Principal Findings

We explored the use of DHTs, including wrist accelerometers, body scales, CGM, and food scales, to remotely monitor the overall well-being of older individuals at risk for frailty and malnutrition. We observed that physical activity endpoints collected at home with a wrist accelerometer and in the lab were significantly different between frailty and nutritional groups. Across all participants, a higher average daily time in SLPA than in MVPA was observed; however, frailty status had a significant effect only on MVPA, with the frail group spending significantly less time in MVPA than the nonfrail group, confirming previous findings [51,52]. A systematic review by Tolley et al [53] reported that time spent in sedentary activity is usually significantly lower in individuals who are frail compared with those who are not. However, the relationship between sedentary time and frailty status is not always consistent [54], and higher sedentary time in nonfrail compared with frail cohorts has also been reported [55]. In this work, sedentary time was significantly higher in the frail compared with the nonfrail group and in the nonrobust compared with the robust group. These findings confirm those reported in our previous study [35], where the frail group spent more time in sedentary activity and less time in MVPA and exhibited significantly a lower 95th percentile of SVMg. In this research, additional physical activity endpoints, including time spent in nonsedentary activity, mean SVMg, and mean activity during M6min and M60min, distinguished frail and nonrobust groups from nonfrail and robust groups. This difference is possibly related to the larger sample size.

A link between nutritional status and physical activity in older adults has already been hypothesized in the literature: Tamamura et al [56] showed that patients with higher MNA-SF scores at admission to rehabilitation wards had greater functional independence at discharge, and Hsueh et al [57] observed that individuals without a healthy diet were more sedentary compared with those with a healthy diet. In this study, we showed that objectively and remotely measured mean activity during M6min and M60min are significantly lower in participants at risk for malnutrition compared with those with normal nutritional status, as assessed with the SNAQ, confirming the relationship between physical activity and nutritional status in older individuals. Notably, physical activity endpoints were not sensitive to MNA-SF groups, suggesting that the SNAQ might be preferable as an anchor metric in studies assessing minimal clinically important differences in digital endpoints.

Wrist-derived physical activity endpoints have already been associated with self-reported behaviors in older individuals [58]; here, we confirmed the relationship between physical activity endpoints and self-reported measures of appetite [59], fatigue [60], and physical function [61]. These findings suggest that digital endpoints collected in free-living settings are concordant with individuals’ perceptions, while unobtrusively providing more objective and sensitive data to complement PROs, as already noted in the literature [37,62]. Indeed, a ceiling effect was observed in self-reported physical function scores but not in physical activity digital endpoints. As noted previously [63], despite the observed correlation, subjective and objective physical activity measures may differ and should potentially be used complementarily [64].

Our findings support the deployment of wrist accelerometers in clinical studies as effective, noninvasive, and highly informative tools for monitoring older adults’ physical activity in free-living conditions and for obtaining insights into their frailty and nutritional status. These tools enable vulnerable populations, such as older individuals, to participate in clinical trials with fewer burdensome visits, thereby expanding access to care. In addition, tracking daily levels of physical activity can help health care professionals assess mobility, sleep, and overall well-being, in turn supporting the development and adjustment of coaching programs for older individuals [65].

Furthermore, we showed that other DHTs can be effectively deployed for older individuals to remotely monitor their weight, body composition, glucose concentration, and meal intake, thus enabling more patient-centric and data-centric clinical trials.

In this work, we showed that the Renpho Body Scale, a small and cost-effective device (~US $30 compared with ~US $7000 for the Tanita scale) that offers scalability and ease of use for both participants and health care professionals, provides accurate measures of weight and percentages of muscle mass, fat mass, fat-free mass, and water mass compared to the research-grade Tanita scale. Although these findings should be further corroborated by comparison against other highly accurate measures of body composition (eg, dual X-ray absorptiometry), they provide evidence for the feasibility of frequent monitoring of weight and body composition in older individuals. These measures can support monitoring underlying conditions, such as AoA, fluid retention, or functional decline, and may be indicative of medication effects [66]. For example, daily body composition measurements can reveal reductions in bone mass, which may indicate osteoporosis, or reductions in muscle mass, which may lead to sarcopenia [67].

Data collected in this study using a digital nutrition scale showed that participants at risk for malnutrition consumed a significantly lower number of daily meals, resulting in lower macronutrient intake, confirming previous findings [68,69]. However, as suggested by Madeira et al [68], this relationship is not absolute: eating more meals does not guarantee good nutritional status if the meals are low in nutritional value. Therefore, a digital nutrition scale represents a useful tool for comprehensive assessment of total dietary intake and nutritional quality in clinical trials. These measures are vital for establishing dietary treatment strategies, tailored to meet individuals’ needs and to prevent energy and nutrient deficiencies, which are associated with increased length of hospital stay, hospital readmission, decreased survival time, and increased incidence of infection in hospitalized older patients [70,71]. Nevertheless, the lower compliance with this device suggests the need for more automated tools to track dietary behavior. For example, CGM data coupled with suitable artificial intelligence algorithms might offer a solution for automatically detecting meal intakes [72].

The importance of monitoring daily glucose fluctuations, including hypoglycemic periods and postprandial peaks, has been increasingly investigated in recent years, and correlations with various microvascular and macrovascular complications have been identified [73]. Although CGM devices are widely used in type 1 diabetes to capture dynamic glycemic profiles, the applicability in older individuals has been less extensively explored. Uotani et al [30] found that women with eating disorders exhibit higher glucose variability and a greater propensity for hypoglycemia, suggesting CGM as a valuable tool for mitigating the risk for severe morbidity and mortality in this population. Zhong et al [74] used CGM for 2 weeks in 698 healthy older individuals (median age 69.1 years), showing excellent glycemic control with median TIR, TBR, and TAR of 94.7%, 3.29%, and 0.64%, respectively. Compared to these values, we observed a higher average TBR, suggesting that older participants at risk for AoA may have poorer glycemic control and may be more susceptible to hypoglycemia.

Understanding older adults’ perceptions of new technologies is crucial for deploying these technologies in clinical trials and everyday life [75,76]. Evaluating the acceptance of DHTs involves measuring both participants’ self-reported usability and acceptability as well as their level of continued use. In this study, more than 80% of participants reported the devices to be mostly to very acceptable to wear/use and indicated that they would wear them for more than 1 week. These findings support the deployment of DHTs for older individuals for longer durations, for example, to assess treatment effects in interventional studies or to track physical and physiological changes over time in longitudinal studies, thereby accessing more granular data than those collected sporadically during in-clinic visits [77]. Compliance was also high: all participants were compliant for more than 90% of monitoring days with the wrist accelerometer, more than 85% with the digital body scale, and more than 60% with the digital nutrition scale. CGM data were available for 43 of 50 participants, who were compliant for more than 80% of monitoring days. These findings support the deployment of DHTs in clinical trials involving older individuals to enhance patient- and data-centricity. Although limited data loss was observed in this study, some issues with CGM and the nutrition scale highlighted the importance of providing clear and detailed training materials to clinical sites for future studies, potentially supported by artificial intelligence [78].

### Limitations

This study has several limitations. First, because it was cross-sectional, causality between digital endpoints, frailty, malnutrition, and other PROs cannot be established. In addition, the sample size across frailty groups was unbalanced, with the frail group accounting for only about 10% of the dataset and all being female. To mitigate this limitation, analyses were also performed by grouping nonfrail and prefrail groups into the nonrobust category and by including sex as a covariate in ANOVA models. Finally, because meal assessment occurred in an unsupervised environment, results may have been affected by unreported or misreported meals.

### Conclusion

In conclusion, monitoring physical activity, weight, and dietary intake represents a cornerstone of effective health management in daily life. As the population ages worldwide, with the number of people aged 60 years and older expected to double by 2050 to approximately 2 billion, understanding the implications of aging becomes crucial for policymakers, health care professionals, and society. In this context, digitalized data collection represents a reliable and cost-effective approach to reducing in-person visits, while providing health care providers with rich longitudinal data on older individuals living in the community or in assisted-living facilities.

## Appendix

Multimedia Appendix 1 Additional information about the Geriatric Anorexia Studies.

## Abbreviations

AoA: anorexia of aging
a.u.: arbitrary units
CGM: continuous glucose monitoring
DHT: digital health technology
FFS: Fried Frailty Score
GAS: Geriatric Anorexia Study
HSD: honestly significant difference
ICC: intraclass correlation coefficient
LoA: limits of agreement
M6min: maximum 6 minutes of activity
M60min: maximum 60 minutes of activity
MNA-SF: Mini Nutrition Assessment – Short Form
MVPA: moderate-to-vigorous physical activity
PRO: patient-reported outcome
PROMIS: Patient-Reported Outcomes Measurement Information System
SLPA: sedentary-to-light physical activity
SNAQ: Simplified Nutritional Appetite Questionnaire
SVMg: signal vector magnitude
TAR: time above range
TBR: time below range
TIR: time in range

## Data Availability

Upon request, and subject to review, the study sponsor, Boston University, will provide data that support the findings of this study. Subject to certain criteria, conditions, and exceptions, Boston University may also provide access to the related individual deidentified participant data. Contact Boston University for more information. Further inquiries can be directed to the corresponding author.

## References

[1] McLaughlin S, Connell C, Heeringa S, Li L, Roberts J (2010). Successful aging in the United States: prevalence estimates from a national sample of older adults. J Gerontol B Psychol Sci Soc Sci.

[2] Maestas N, Mullen KJ, Powell D (2023). The effect of population aging on economic growth, the labor force, and productivity. Am Econ J Macroecon.

[3] Landi F, Calvani R, Tosato M, Martone AM, Ortolani E, Savera G, Sisto A, Marzetti E (2016). Anorexia of aging: risk factors, consequences, and potential treatments. Nutrients.

[4] Aprahamian I, Coats A, Morley J, Klompenhouwer T, Anker SD, International Advisory Board, and Regional Advisory Boards for North America, Latin America, Europe and Japan (2023). Anorexia of aging: an international assessment of healthcare providers' knowledge and practice gaps. J Cachexia Sarcopenia Muscle.

[5] Perera LAM, Chopra A, Shaw AL (2021). Approach to patients with unintentional weight loss. Med Clin North Am.

[6] AlFehaidi A, Khan S, Abdelrahman R, Ahel NT, Shine P, De Ramos MD, Skairjeh NM, Khan SA, Al-Saadi RK (2024). Predictors of malnutrition among older residents in Qatari long-term care facilities: a retrospective study. BMC Nutr.

[7] Ono S, Sasabuchi Y, Yamana H, Yokota I, Okada A, Matsui H, Itai S, Yonenaga K, Tonosaki K, Watanabe R, Ono Y, Yasunaga H, Hoshi K (2024). Weight loss and functional decline in older Japanese people: a cohort study using large-scale claims data. Arch Gerontol Geriatr.

[8] Hoogendijk E, van Kan GA, Guyonnet S, Vellas B, Cesari M (2015). Components of the frailty phenotype in relation to the frailty index: results from the Toulouse frailty platform. J Am Med Dir Assoc.

[9] Fried LP, Tangen CM, Walston J, Newman AB, Hirsch C, Gottdiener J, Seeman T, Tracy R, Kop WJ, Burke G, McBurnie MA, Cardiovascular Health Study Collaborative Research Group (2001). Frailty in older adults: evidence for a phenotype. J Gerontol A Biol Sci Med Sci.

[10] Takayanagi N, Sudo M, Yamashiro Y, Lee S, Kobayashi Y, Niki Y, Shimada H (2019). Relationship between daily and in-laboratory gait speed among healthy community-dwelling older adults. Sci Rep.

[11] Czech M, Psaltos D, Zhang H, Adamusiak T, Calicchio M, Kelekar A, Messere A, Van Dijk KRA, Ramos V, Demanuele C, Cai X, Santamaria M, Patel S, Karahanoglu FI (2020). Age and environment-related differences in gait in healthy adults using wearables. NPJ Digit Med.

[12] McCambridge J, Witton J, Elbourne D (2014). Systematic review of the Hawthorne effect: new concepts are needed to study research participation effects. J Clin Epidemiol.

[13] Batsis J, DiMilia P, Seo L, Fortuna KL, Kennedy MA, Blunt HB, Bagley PJ, Brooks J, Brooks E, Kim SY, Masutani RK, Bruce ML, Bartels SJ (2019). Effectiveness of ambulatory telemedicine care in older adults: a systematic review. J Am Geriatr Soc.

[14] Ní Scanaill C, Carew S, Barralon P, Noury N, Lyons D, Lyons G (2006). A review of approaches to mobility telemonitoring of the elderly in their living environment. Ann Biomed Eng.

[15] Cordeiro JV (2021). Digital technologies and data science as health enablers: an outline of appealing promises and compelling ethical, legal, and social challenges. Front Med (Lausanne).

[16] Kim B, Hunt M, Muscedere J, Maslove DM, Lee J (2021). Using consumer-grade physical activity trackers to measure frailty transitions in older critical care survivors: exploratory observational study. JMIR Aging.

[17] Zhang X, Feng L, Munster B, Hobbelen H, Lamoth CJ (2024). Distinguishing (pre)frail from non-frail older adults based on walking pattern: a scoping review on gait parameters derived from inertial sensors. Gait Posture.

[18] Murphy RA, Patel KV, Kritchevsky SB, Houston DK, Newman AB, Koster A, Simonsick EM, Tylvasky FA, Cawthon PM, Harris TB (2014). Weight change, body composition, and risk of mobility disability and mortality in older adults: a population-based cohort study. J Am Geriatr Soc.

[19] Ulugerger Avci G, Bektan Kanat B, Can G, Suzan V, Unal D, Degirmenci P, Avci S, Yavuzer H, Erdincler DS, Doventas A (2023). The impact of sarcopenia and obesity on mortality of older adults: five years results. Ir J Med Sci.

[20] Rudzińska A, Piotrowicz K, Perera I, Gryglewska B, Gąsowski J (2023). Poor appetite in frail older persons-a systematic review. Nutrients.

[21] Cox N, Morrison L, Ibrahim K, Robinson S, Sayer A, Roberts H (2020). New horizons in appetite and the anorexia of ageing. Age Ageing.

[22] Gauthier A, Jaunzarins B, MacDougall S-J, Laurence M, Kabaroff JL, Godwin AA, Dorman SC (2013). Evaluating the reliability of assessing home-packed food items using digital photographs and dietary log sheets. J Nutr Educ Behav.

[23] Tanweer A, Khan S, Mustafa F, Imran S, Humayun A, Hussain Z (2022). Improving dietary data collection tools for better nutritional assessment – a systematic review. Comput Methods Programs Biomed Update.

[24] Gariballa S, Forster S (2008). Dietary intake of older patients in hospital and at home: the validity of patient kept food diaries. J Nutr Health Aging.

[25] Kalinowska K, Wojnowski W, Tobiszewski M (2021). Smartphones as tools for equitable food quality assessment. Trends Food Sci Technol.

[26] Iizuka K, Deguchi K, Ushiroda C, Yanagi K, Seino Y, Suzuki A, Yabe D, Sasaki H, Sasaki S, Saitoh E, Naruse H (2024). A study on the compatibility of a food-recording application with questionnaire-based methods in healthy Japanese individuals. Nutrients.

[27] Theodore Armand TP, Kim H-C, Kim J-I (2024). Digital anti-aging healthcare: an overview of the applications of digital technologies in diet management. J Pers Med.

[28] Ryu J, Kim S, Lim Y, Ohn JH, Kim S, Cho JH, Park HS, Lee J, Kim ES, Kim N, Song JE, Kim SH, Suh E, Mukhtorov D, Park JH, Kim SK, Kim HW (2024). Sodium intake estimation in hospital patients using AI-based imaging: prospective pilot study. JMIR Form Res.

[29] Galindo R, Aleppo G (2020). Continuous glucose monitoring: the achievement of 100 years of innovation in diabetes technology. Diabetes Res Clin Pract.

[30] Uotani N, Noma S, Akamine M, Miyawaki T (2022). Continuous glucose monitoring for detection of glycemic variability, hypoglycemia, and hyperglycemia in women with eating disorders. Biopsychosoc Med.

[31] Hegedus E, Salvy S-J, Wee CP, Naguib M, Raymond JK, Fox DS, Vidmar AP (2021). Use of continuous glucose monitoring in obesity research: a scoping review. Obes Res Clin Pract.

[32] Presseller EK, Parker MN, Lin M, Weimer J, Juarascio AS (2020). The application of continuous glucose monitoring technology to eating disorders research: an idea worth researching. Int J Eat Disord.

[33] Lupoli R, Lembo E, Ciciola P, Schiavo L, Pilone V, Capaldo B (2020). Continuous glucose monitoring in subjects undergoing bariatric surgery: diurnal and nocturnal glycemic patterns. Nutr Metab Cardiovasc Dis.

[34] Sadıç A, Şahiner Z, Eşme M, Balcı C, Doğu BB, Cankurtaran M, Gülhan Halil M (2025). Malnutrition and frailty as independent predictors of adverse outcomes in hospitalized older adults: a prospective single center study. Medicina (Kaunas).

[35] Camerlingo N, Shaafi Kabiri N, Psaltos DJ, Kelly M, Wicker MK, Messina I, Auerbach SH, Zhang H, Messere A, Isik Karahanoglu F, Santamaria M, Demanuele C, Caouette D, Thomas KC (2024). Monitoring gait and physical activity of elderly frail individuals in free-living environment: a feasibility study. Gerontology.

[36] Omran M, Morley J (2000). Assessment of protein energy malnutrition in older persons, part I: history, examination, body composition, and screening tools. Nutrition.

[37] McDermott MM, Liu K, O'Brien E, Guralnik JM, Criqui MH, Martin GJ, Greenland P (2000). Measuring physical activity in peripheral arterial disease: a comparison of two physical activity questionnaires with an accelerometer. Angiology.

[38] Winter J, Flanagan D, McNaughton SA, Nowson C (2013). Nutrition screening of older people in a community general practice, using the MNA-SF. J Nutr Health Aging.

[39] Wang T, Shen J (2018). Usefulness of Simplified Nutritional Appetite Questionnaire (SNAQ) in appetite assessment in elder patients with liver cirrhosis. J Nutr Health Aging.

[40] Acar Tek N, Karaçil-Ermumcu MŞ (2018). Determinants of health related quality of life in home dwelling elderly population: appetite and nutritional status. J Nutr Health Aging.

[41] Rolland Y, Perrin A, Gardette V, Filhol N, Vellas B (2012). Screening older people at risk of malnutrition or malnourished using the Simplified Nutritional Appetite Questionnaire (SNAQ): a comparison with the Mini-Nutritional Assessment (MNA) tool. J Am Med Dir Assoc.

[42] Perfetto EM, Burke L, Oehrlein EM, Epstein RS (2015). Patient-focused drug development: a new direction for collaboration. Med Care.

[43] Terwee C, Crins M, Roorda L, Cook KF, Cella D, Smits N, Schalet BD (2021). International application of PROMIS computerized adaptive tests: US versus country-specific item parameters can be consequential for individual patient scores. J Clin Epidemiol.

[44] Erb MK, Karlin DR, Ho BK, Thomas KC, Parisi F, Vergara-Diaz GP, Daneault J-F, Wacnik PW, Zhang H, Kangarloo T, Demanuele C, Brooks CR, Detheridge CN, Shaafi Kabiri N, Bhangu JS, Bonato P (2020). mHealth and wearable technology should replace motor diaries to track motor fluctuations in Parkinson's disease. NPJ Digit Med.

[45] Mahadevan N, Christakis Y, Di J, Bruno J, Zhang Y, Dorsey ER, Pigeon WR, Beck LA, Thomas K, Liu Y, Wicker M, Brooks C, Kabiri NS, Bhangu J, Northcott C, Patel S (2021). Development of digital measures for nighttime scratch and sleep using wrist-worn wearable devices. NPJ Digit Med.

[46] Howell J, Strong B, Weisenberg J, Kakade A, Gao Q, Cuddihy P, Delisle S, Kachnowski S, Maurer MS (2010). Maximum daily 6 minutes of activity: an index of functional capacity derived from actigraphy and its application to older adults with heart failure. J Am Geriatr Soc.

[47] Saville DJ (1990). Multiple comparison procedures: the practical solution. Am Stat.

[48] Cicchetti D, Bronen R, Spencer S, Haut S, Berg A, Oliver P, Tyrer P (2006). Rating scales, scales of measurement, issues of reliability: resolving some critical issues for clinicians and researchers. J Nerv Ment Dis.

[49] Troiano R, McClain J, Brychta R, Chen K (2014). Evolution of accelerometer methods for physical activity research. Br J Sports Med.

[50] Lohne-Seiler H, Hansen B, Kolle E, Anderssen S (2014). Accelerometer-determined physical activity and self-reported health in a population of older adults (65-85 years): a cross-sectional study. BMC Public Health.

[51] Kikuchi H, Inoue S, Amagasa S, Fukushima N, Machida M, Murayama H, Fujiwara T, Chastin S, Owen N, Shobugawa Y (2021). Associations of older adults' physical activity and bout-specific sedentary time with frailty status: compositional analyses from the NEIGE study. Exp Gerontol.

[52] Razjouyan J, Naik AD, Horstman MJ, Kunik ME, Amirmazaheri M, Zhou H, Sharafkhaneh A, Najafi B (2018). Wearable sensors and the assessment of frailty among vulnerable older adults: an observational cohort study. Sensors (Basel).

[53] Tolley APL, Ramsey KA, Rojer AGM, Reijnierse EM, Maier AB (2021). Objectively measured physical activity is associated with frailty in community-dwelling older adults: a systematic review. J Clin Epidemiol.

[54] Park K-N, Kim S-H (2022). Consumer wearable device-based measures of physical activity and energy expenditure in community-dwelling older adults with different levels of frailty: a STROBE compliant study. Medicine (Baltimore).

[55] Chen S, Chen T, Kishimoto H, Yatsugi H, Kumagai S (2020). Associations of objectively measured patterns of sedentary behavior and physical activity with frailty status screened by the Frail Scale in Japanese community-dwelling older adults. J Sports Sci Med.

[56] Tamamura Y, Hachiuma C, Matsuura M, Shiba S, Nishikimi T (2024). Relationship between improvement in physical activity and three nutritional assessment indicators in patients admitted to a convalescent rehabilitation ward. Nutrients.

[57] Hsueh M-C, Rutherford R, Huang Y-H, Chang Chien H-Y, Chang C-H, Park J-H, Liao Y (2019). Are older adults without a healthy diet less physically active and more sedentary?. Nutrients.

[58] Maes I, Mertens L, Poppe L, Vetrovsky T, Crombez G, De Backere F, Brondeel R, Van Dyck D (2023). Within-person associations of accelerometer-assessed physical activity with time-varying determinants in older adults: time-based ecological momentary assessment study. JMIR Aging.

[59] Tsai L-T, Boyle E, Buhl SF, Kock G, Brønd JC, Visser M, Mendonça Nno, Shiroma EJ, Caserotti P (2023). Associations between appetite, physical activity and sedentary behaviour from hip- and wrist-worn accelerometers in community-dwelling older adults. Geriatr Gerontol Int.

[60] Zaslavsky O, Su Y, Rillamas-Sun E, Roopsawang I, LaCroix AZ (2020). Accelerometer-measured physical activity levels and fatigue in older women. J Aging Phys Act.

[61] Barone Gibbs B, Sternfeld B, Whitaker KM, Brach JS, Hergenroeder AL, Jacobs DR, Reis JP, Sidney S, White D, Pettee Gabriel K (2021). Bidirectional associations of accelerometer-derived physical activity and stationary behavior with self-reported mental and physical health during midlife. Int J Behav Nutr Phys Act.

[62] Oyeyemi A, Umar M, Oguche F, Aliyu S, Oyeyemi A (2014). Accelerometer-determined physical activity and its comparison with the International Physical Activity Questionnaire in a sample of Nigerian adults. PLoS One.

[63] Vetter V, Özince DD, Kiselev J, Düzel S, Demuth I (2023). Self-reported and accelerometer-based assessment of physical activity in older adults: results from the Berlin Aging Study II. Sci Rep.

[64] Yang Y, Hirdes JP, Dubin JA, Lee J (2019). Fall risk classification in community-dwelling older adults using a smart wrist-worn device and the resident assessment instrument-home care: prospective observational study. JMIR Aging.

[65] Foong YC, Chherawala N, Aitken D, Scott D, Winzenberg T, Jones G (2016). Accelerometer-determined physical activity, muscle mass, and leg strength in community-dwelling older adults. J Cachexia Sarcopenia Muscle.

[66] Saxon SV, Etten MJ, Perkins EA (2021). Physical Change and Aging: A Guide for Helping Professions.

[67] Laurent M, Dedeyne L, Dupont J, Mellaerts B, Dejaeger M, Gielen E (2019). Age-related bone loss and sarcopenia in men. Maturitas.

[68] Madeira T, Severo M, Correia D, Lopes C, Gorjão Clara J (2022). Nutritional intake and malnutrition in institutionalised and non-institutionalised older adults. Br J Nutr.

[69] Ozer NT, Akin S, Gunes Sahin G, Sahin S (2022). Prevalence of malnutrition diagnosed by the Global Leadership Initiative on Malnutrition and Mini Nutritional Assessment in older adult outpatients and comparison between the Global Leadership Initiative on Malnutrition and Mini Nutritional Assessment energy-protein intake: a cross-sectional study. JPEN J Parenter Enteral Nutr.

[70] Abugroun A, Nayyar A, Abdel-Rahman M, Patel P (2021). Impact of malnutrition on hospitalization outcomes for older adults admitted for sepsis. Am J Med.

[71] Chen C, Schilling L, Lyder C (2001). A concept analysis of malnutrition in the elderly. J Adv Nurs.

[72] van den Brink WJ, van den Broek TJ, Palmisano S, Wopereis S, de Hoogh IM (2022). Digital biomarkers for personalized nutrition: predicting meal moments and interstitial glucose with non-invasive, wearable technologies. Nutrients.

[73] Suh S, Kim JH (2015). Glycemic variability: how do we measure it and why is it important?. Diabetes Metab J.

[74] Zhong H, Zhang K, Lin L, Yan Y, Shen L, Chen H, Liang X, Chen J, Miao Z, Zheng J-S, Chen Y-M (2024). Two-week continuous glucose monitoring-derived metrics and degree of hepatic steatosis: a cross-sectional study among Chinese middle-aged and elderly participants. Cardiovasc Diabetol.

[75] Lu J, Wang W, Goh J, Maier A (2024). A practical guide for selecting continuous monitoring wearable devices for community-dwelling adults. Heliyon.

[76] Garcia Reyes EP, Kelly R, Buchanan G, Waycott J (2023). Understanding older adults’ experiences with technologies for health self-management: interview study. JMIR Aging.

[77] Hulleck A, Menoth Mohan D, Abdallah N, El Rich M, Khalaf K (2022). Present and future of gait assessment in clinical practice: towards the application of novel trends and technologies. Front Med Technol.

[78] Jayakumar P, Moore MG, Furlough KA, Uhler LM, Andrawis JP, Koenig KM, Aksan N, Rathouz PJ, Bozic KJ (2021). Comparison of an artificial intelligence–enabled patient decision aid vs educational material on decision quality, shared decision-making, patient experience, and functional outcomes in adults with knee osteoarthritis: a randomized clinical trial. JAMA Netw Open.

