# Supplementary material for: Leveraging Digital Health Technologies to Assess Older Adults’ Frailty and Nutritional Status: Two Cross-Sectional Studies
Source: JMIR Aging. 2026 Apr 17;9:e77816. doi: 10.2196/77816 (PMC13135171; doi:10.2196/77816)
Supplement: Multimedia Appendix 1 [file aging_v9i1e77816_app1.docx]

## **SUPPLEMENTAL MATERIAL**


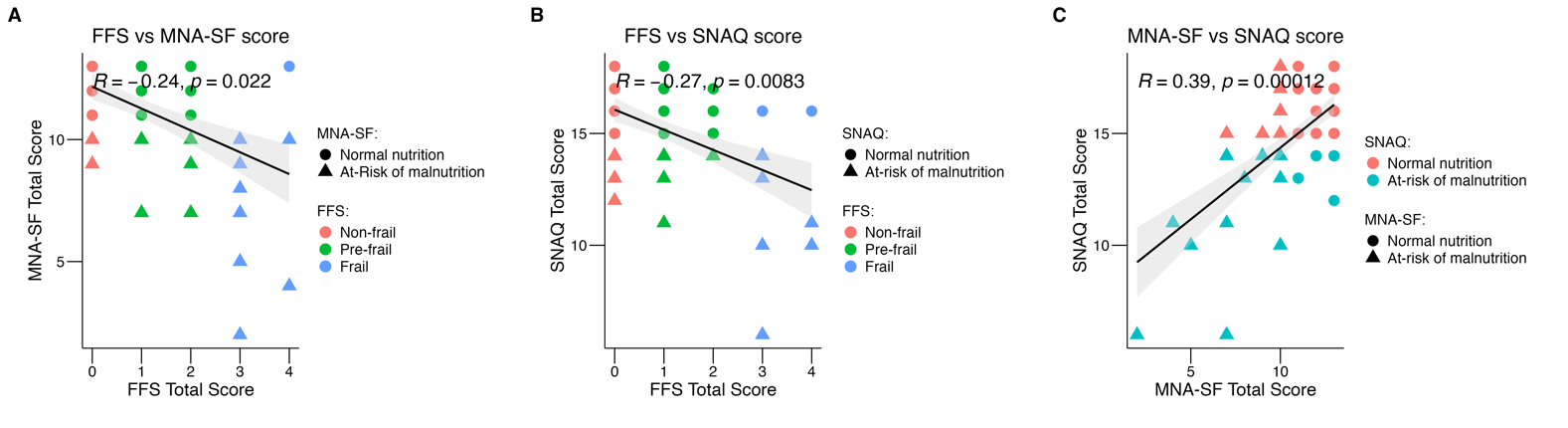


Supplemental Figure 1. Relationship between FFS, MNA-SF, and SNAQ. The relationship between FFS and MNA-SF (panel A), FFS and SNAQ (panel B), MNA-SF and SNAQ (panel C) is assessed via scatter plots with regression line (black) and 95% confidence interval (gray shaded area). Dots colors represent FFS in panels A and B (red: non-frail, green: pre-frail, blue: frail), and SNAQ in panel C (pink: normal nutrition, cyan: at-risk of malnutrition). Dots shapes represent MNA-SF in panels A and C (round: normal nutrition, triangle: at-risk of malnutrition), and SNAQ in panel B (round: normal nutrition, triangle: at-risk of malnutrition). Spearman’s correlation coefficients with p-values are reported in each panel.

**Renpho Body Scale can reliably measure weight and muscle mass**

Two participants did not perform all three weight assessments in-clinic: one was unwilling to perform the last visit assessments, and the other could not use Tanita in any of the visits because of lack of mobility in arm and hand. ICC and Pearson’s R showed excellent agreement and strong correlation for all body composition endpoints, except bone mass. Notably, perfect agreement and correlation were observed for weight. Mean percent errors lower than 1% were found for weight and water mass, and lower than 4% for muscle mass, fat mass and fat-free mass.

The agreement and error metrics for the weight and percent body composition endpoints measured from Renpho Body Scale and Tanita used in-clinic are summarized in the Supplemental Table 1.

Bland-Altman plots and scatter plots are depicted in Supplemental Figure 2, for weight and muscle mass, and in the Supplemental Figure 3 for the other endpoints. Compared to Tanita, Renpho Body Scale slightly overestimated weight and underestimated muscle mass (mean bias of 0.246 Kg and -1.552%, respectively). Only for water mass, the 0-threshold falls within the 95% confidence interval of the bias. Bias was homogeneous throughout the measurement range for all endpoints. Supplemental Figure 2C also shows the presence of an outlier, whose average muscle mass is 63.06 Kg, estimated by Tanita, and 39.50 Kg, estimated by Renpho Body Scale. This participant had a BMI of 50.4 Kg/m^2^ (and weighed 141.63 Kg, as measured by Tanita), thus falling under Class III obesity, according to the US Centers for Disease Control and Prevention (CDC) [1]. For this class of participants, BIA methods are known for having poor reliability to assess body composition, thus explaining the presence of such outlier [2] (the same participant is an outlier for other body composition endpoints). This participant had a BMI of 50.4 Kg/m^2^ (and weighed 141.63 Kg, as measured by Tanita), thus falling under Class III obesity, according to the US Centers for Disease Control and Prevention (CDC) [1]. For this class of participants, BIA methods are known for having poor reliability to assess body composition, thus explaining the presence of such outlier [2] (the same participant is an outlier for other body composition endpoints).

Results considering body composition measurements expressed as mass values (Kg, rather than percentages) are reported in Supplemental Figure 4 and Supplemental Table 2. ICC showed excellent agreement (always higher than that observed with percent values) for all body composition endpoints, including bone mass. However, higher mean percent error and non-homogeneous bias were observed for muscle mass, fat-free mass, and bone mass, leading to higher errors for lower and higher measurements. This data suggests that percent body composition measures should be preferred to absolute body composition measures for this device.


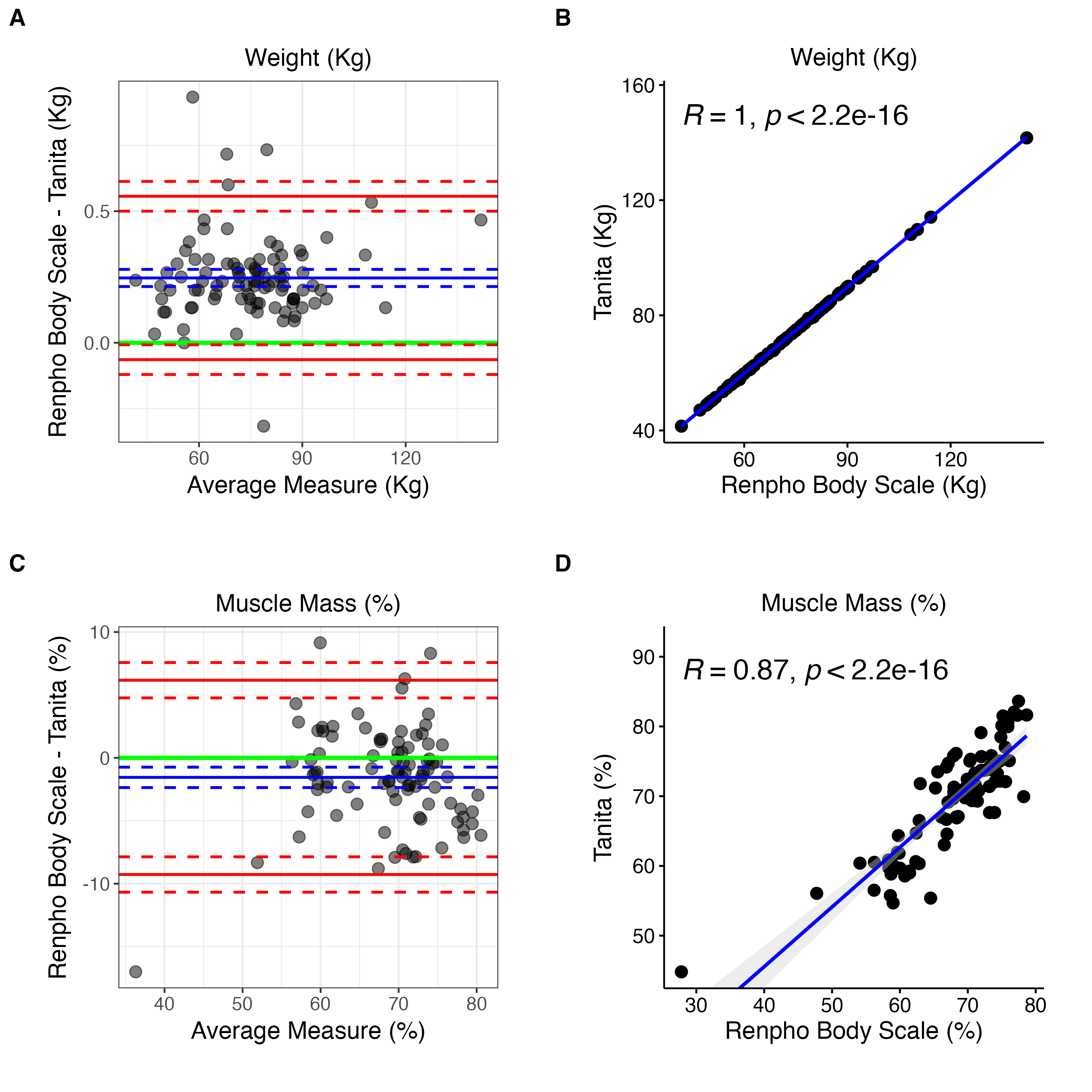


Supplemental Figure 2. Assessment of Renpho Body Scale against Tanita for weight (panels A, B) and percentage of muscle mass (panels C, D) measured in-clinic. Bland-Altman plots (panels A, C) show a mean bias (blue solid line, computed as Renpho Body Scale – Tanita) equal to 0.246 Kg for weight, and equal to -1.552 % for muscle mass, homogeneous throughout the measurement range. Red solid lines represent the LoA. Corresponding 95% confidence intervals are in dashed lines, while green solid line represents the 0-threshold. Scatter plots (panels B, D) show highly correlated measurements for both the endpoints. Regression line (solid blue line) with 95% confidence interval (shaded gray area) are depicted, and the Pearson’s R with the related p-values are annotated in the figure. (LoA: Limit of Agreement)

Supplemental Table 1. Bias with LoA, mean absolute difference, mean percent error, ICC with LB and UB, and Pearson’s R with p-value computed between weight, and percentages of muscle mass, fat mass, fat-free mass, bone mass, and water mass, collected in-clinic from Renpho Body Scale (test device) and Tanita (reference device). Error metrics are computed as difference between meaasurements collected from Renpho Body Scale and those collected from Tanita. (LoA: Limits of Agreement, MAD: Mean absolute difference, MPE: Mean percent error, ICC: intra-class correlation coefficient, LB: Lower bound, UB: Upper bound).

| Endpoint | N | Bias (LoA) | MAD | MPE, % | ICC  (LB, UB) | Pearson’s R (p-value) |
| --- | --- | --- | --- | --- | --- | --- |
| Weight (Kg) | 93 | 0.246  (-0.064, 0.557) | 0.253 | 0.342 | 1.000  (0.994, 1.00) | 1.000  (<0.001) |
| Muscle mass (%) | 93 | -1.552  (-9.273, 6.169) | 3.164 | -2.733 | 0.848  (0.752, 0.904) | 0.866  (<0.001) |
| Fat Mass (%) | 93 | 1.110  (-7.036, 9.257) | 3.205 | 3.682 | 0.853  (0.781, 0.901) | 0.861  (<0.001) |
| Fat-Free Mass (%) | 93 | -1.152  (-9.291, 6.988) | 3.254 | -2.027 | 0.851  (0.778, 0.901) | 0.964  (<0.001) |
| Bone Mass (%) | 93 | 0.252  (-0.565, 1.070) | 0.385 | 5.717 | 0.394  (0.131, 0.588) | 0.473  (<0.001) |
| Water Mass (%) | 93 | 0.177  (-6.577, 6.931) | 2.666 | -0.379 | 0.820  (0.741, 0.876) | 0.834  (<0.001) |

Supplemental Table 2. Bias with LoA, mean absolute difference, mean percent error, ICC with LB and UB, and Pearson’s R with p-value computed between muscle mass, fat mass, fat-free mass, bone mass, and water mass, collected in-clinic from Renpho Body Scale (test device) and Tanita (reference device). Error metrics are computed as difference between measurements collected from Renpho Body Scale and those collected from Tanita. (LoA: Limits of Agreement, MAD: Mean absolute difference, MPE: Mean percent error, ICC: intra-class correlation coefficient, LB: Lower bound, UB: Upper bound)

| Endpoint | N | Bias (LoA) | MAD | MPE, % | ICC  (LB, UB) | Pearson’s r (p-value) |
| --- | --- | --- | --- | --- | --- | --- |
| Muscle mass (Kg) | 93 | -0.673  (-7.817, 6.472) | 2.297 | -1.094 | 0.942  (0.914, 0.961) | 0.950  (<0.001) |
| Fat Mass (Kg) | 93 | 1.659  (-6.979, 9.297) | 2.716 | 6.052 | 0.915  (0.850, 0.949) | 0.948  (<0.001) |
| Fat-Free Mass (Kg) | 93 | -0.373  (-7.785, 7.039) | 2.443 | -0.398 | 0.944  (0.917, 0.962) | 0.952  (<0.001) |
| Bone Mass (Kg) | 93 | 0.186  (-0.389, 0.760) | 0.274 | 7.206 | 0.759  (0.501, 0.870) | 0.897  (<0.001) |
| Water Mass (Kg) | 93 | 0.511  (-5.694, 6.716) | 2.209 | -0.986 | 0.925  (0.889, 0.949) | 0.925  (<0.001) |


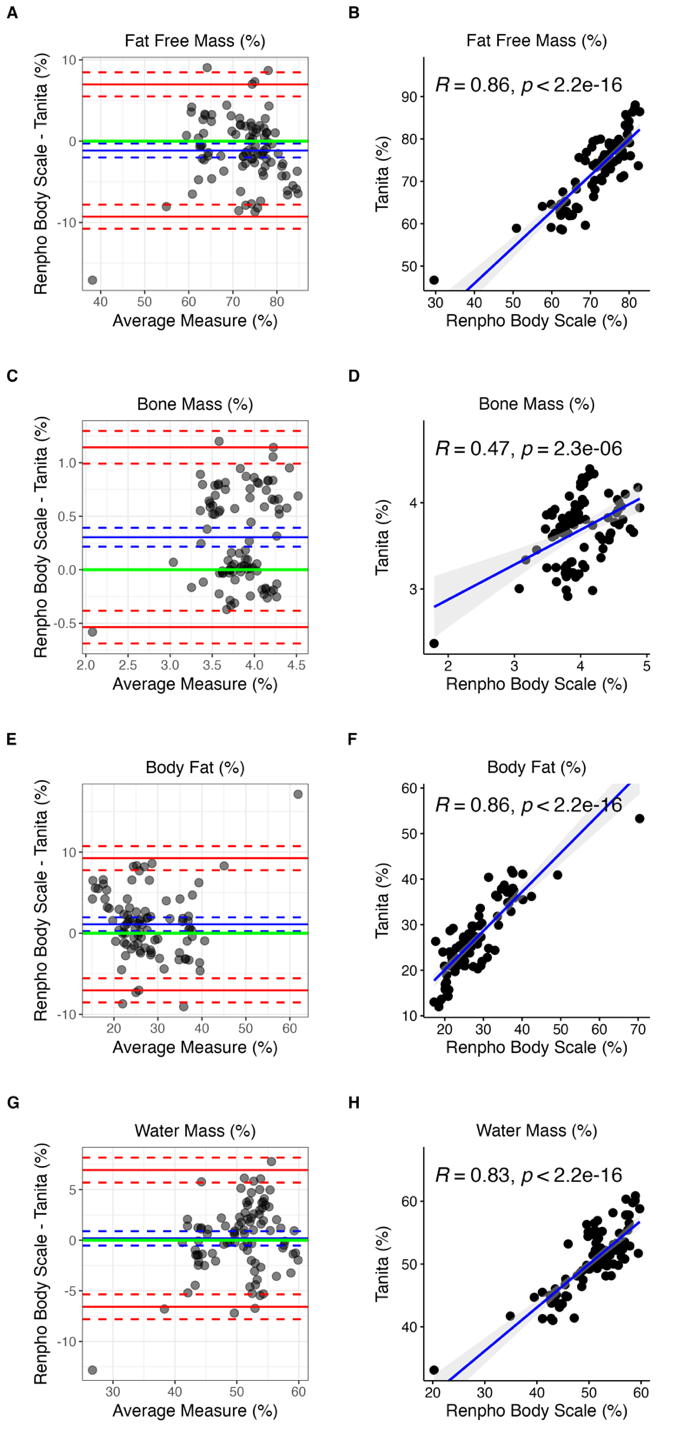


Supplemental Figure 3. Assessment of Renpho Body Scale against Tanita for percentages of fat-free mass (panels A, B), bone mass (panels C, D), fat mass (panels E, F), and water mass (panels G, H), measured in-clinic. Bland-Altman plots (panels A, C, E, G) show the mean bias (blue solid line, computed as Renpho Body Scale – Tanita) with LoA (red solid lines). Corresponding 95% confidence intervals are in dashed lines, while green solid line represents the 0-threshold. Scatter plots (panels B, D, F, H) show highly correlated measurements for all the endpoints. Regression line (solid blue line) with 95% confidence interval (shaded gray area) are depicted, and the Pearson’s R with the related p-value are annotated in the figure. (LoA: Limit of Agreement)


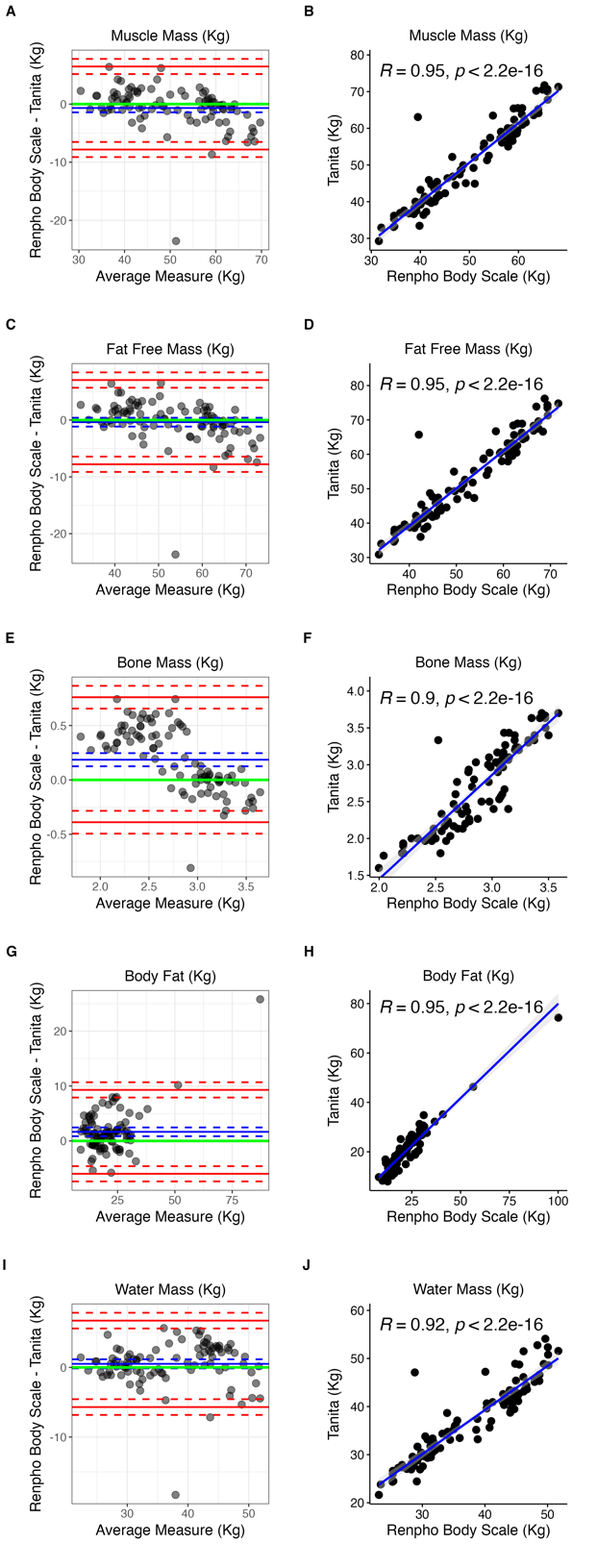


Supplemental Figure 4. Assessment of Renpho Body Scale against Tanita for muscle mass (panels A, B), fat-free mass (panels C, D), bone mass (panels E, F), fat mass (panels G, H), and water mass (panels I, J), measured in-clinic. Bland-Altman plots (panels A, C, E, G) show the mean bias (blue solid line, computed as Renpho Body Scale – Tanita) with LoA (red solid lines). Corresponding 95% confidence intervals are in dashed lines, while green solid line represents the 0-threshold. Scatter plots (panels B, D, F, H) show highly correlated measurements for all the endpoints. Regression line (solid blue line) with 95% confidence interval (shaded gray area) are depicted, and the Pearson’s R with the related p-value are annotated in the figure. (LoA: Limit of Agreement)

Supplemental Table 3. Digital endpoints, averaged across all participants, and across frailty and robustness groups. Values are reported as mean±sd.

| DHT/Digital endpoint | Overall | Non-frail/ Robust | Pre-frail | Frail | Non-Robust |
| --- | --- | --- | --- | --- | --- |
|  |  |  |  |  |  |
| Renpho Body Scale | N=94 | N=39 | N=45 | N=10 | N=55 |
| Weight (Kg) | 72.45±16.5 | 72.73±13.0 | 73.03±14.4 | 68.84±32.4 | 72.25±18.7 |
| Muscle mass (%) | 68.77±7.94 | 69.91±5.94 | 68.89±7.17 | 63.85±14.63 | 67.92±9.12 |
| Fat Mass (%) | 27.24±8.21 | 26.05±6.06 | 27.18±7.33 | 32.07±15.6 | 28.12±9.48 |
| Fat-Free Mass (%) | 72.76±8.22 | 73.95±6.06 | 72.81±7.34 | 67.92±15.6 | 71.87±9.48 |
| Bone Mass (%) | 3.994±0.43 | 4.041±0.33 | 3.931±0.32 | 4.075±0.94 | 3.959±0.49 |
| Water Mass (%) | 61.26±6.51 | 52.16±5.14 | 51.54±6.10 | 46.60±10.7 | 50.59±7.35 |
| CGM | N=43 | N=19 | N=19 | N=5 | N=24 |
| Mean (mg/dL) | 85.74±6.86 | 86.69±6.51 | 85.41±6.71 | 83.39±9.48 | 84.99±7.18 |
| SD (mg/dL) | 16.49±4.51 | 16.56±4.54 | 16.69±4.81 | 15.44±3.87 | 16.43±4.58 |
| Minimum (mg/dL) | 61.86±7.23 | 62.68±6.06 | 61.66±7.09 | 59.45±12.1 | 61.20±8.10 |
| Maximum (mg/dL) | 139.2±17.1 | 141.3±15.8 | 139.1±17.7 | 131.8±21.3 | 137.5±18.3 |
| TIR (%) | 85.10±14.4 | 86.97±11.9 | 85.20±14.5 | 77.66±22.6 | 83.63±16.3 |
| TBR (%) | 12.94±12.2 | 11.41±10.5 | 12.79±12.2 | 19.33±19.1 | 14.16±13.7 |
| TAR (%) | 0.215±0.48 | 0.141±0.21 | 0.333±0.67 | 0.052±0.12 | 0.274±061 |
| Renpho Nutrition Scale/iPhone | N=50 | N=21 | N=23 | N=6 | N=29 |
| Number of daily meals | 3.049±0.99 | 3.178±1.22 | 2.98±0.66 | 2.85±1.30 | 2.955±0.81 |
| Meal duration (min) | 32.45±16.4 | 34.59±19.3 | 29.87±13.1 | 34.83±17.9 | 30.89±14.0 |
| Time between consecutive meals (hr) | 4.93±1.34 | 5.06±1.51 | 4.83±0.93 | 4.84±2.16 | 4.83±1.23 |
| Meals’ net weight (g) | 291.5±81.1 | 315.6±86.9 | 290.8±68.4 | 210.0±57.2 | 274.1±73.3 |
| Meals’ net energy (kCal) | 356.9±101 | 368.7±104 | 374.6±79.0 | 248.0±117 | 348.4±100 |
| Meals’ net fat (g) | 15.78±4.61 | 16.20±4.99 | 16.59±3.47 | 11.22±5.21 | 15.48±4.38 |
| Meals’ net sodium (mg)^a^ | 323.1±102 | 333.8±95.0 | 345.7±77.4 | 198.5±134 | 315.2±107 |
| Meals’ net potassium (mg)^a^ | 530.2±159 | 550.2±146 | 562.5±127 | 335.9±198 | 515.7±168 |
| Meals’ net carbohydrates (g) | 32.15±8.66 | 32.97±8.37 | 33.30±8.53 | 24.84±7.85 | 31.55±8.97 |
| Meals’ net protein (g) | 22.44±6.70 | 23.14±6.77 | 23.74±5.07 | 14.99±8.23 | 21.93±6.73 |
| ^a^ significantly different across frailty groups ^b^ significantly different across robustness groups | | | | | |

Supplemental Table 4. Digital endpoints averaged across all participants, and across nutritional groups, as assessed by MNA-SF and SNAQ. Values are reported as mean±sd.

| DHT/Digital endpoints | MNA-SF | | SNAQ | |
| --- | --- | --- | --- | --- |
|  | Normal nutrition | At-risk of malnutrition | Normal nutrition | At-risk of malnutrition |
| Renpho Body Scale | N=67 | N=27 | N=70 | N=24 |
| Weight (Kg) | 74.30±13.3 | 67.69±22.3 | 72.51±13.7 | 72.28±23.0 |
| Muscle mass (%) | 68.95±6.57 | 69.06±10.9 | 67.93±6.68 | 66.03±11.0 |
| Fat Mass (%) | 27.11±6.68 | 27.58±11.5 | 26.95±6.82 | 28.08±11.5 |
| Fat-Free Mass (%) | 72.89±6.69 | 72.41±11.5 | 73.05±6.83 | 71.91±11.5 |
| Bone Mass (%) | 3.944±0.29 | 4.127±0.66 | 4.00±0.33 | 3.98±0.65 |
| Water Mass (%) | 51.56±5.68 | 50.47±8.39 | 51.51±5.71 | 50.54±8.55 |
| CGM | N=32 | N=11 | N=33 | N=10 |
| Mean (mg/dL) | 85.81±6.81 | 85.55±7.34 | 86.22±6.13 | 84.15±9.10 |
| SD (mg/dL) | 16.29±4.50 | 17.07±4.72 | 16.38±4.38 | 16.82±5.17 |
| Minimum (mg/dL) | 62.04±7.03 | 61.31±8.11 | 62.97±6.70 | 58.19±8.03 |
| Maximum (mg/dL) | 138.6±16.0 | 141.0±20.7 | 140.0±15.6 | 136.5±22.2 |
| TIR (%) | 85.68±14.0 | 83.41±16.2 | 86.98±12.7 | 78.90±18.5 |
| TBR (%) | 12.48±12.0 | 14.29±13.7 | 11.37±10.9 | 18.12±15.7 |
| TAR (%) | 0.196±0.50 | 0.272±0.40 | 0.264±0.53 | 0.054±0.12 |
| Renpho Nutrition Scale/iPhone | N=38 | N=12 | N=39 | N=11 |
| Number of daily meals* | 2.910±0.75 | 3.487±1.49 | 3.24±0.99 | 2.36±0.67 |
| Meal duration (min) | 30.72±15.0 | 37.90±19.8 | 31.17±14.38 | 36.97±22.4 |
| Time between consecutive daily meals (hr) | 5.10±1.17 | 4.38±1.72 | 4.72±1.32 | 5.72±1.18 |
| Meals’ net weight (g)* | 293.1±73.9 | 286.6±104 | 305.9±79.9 | 240.6±65.8 |
| Meals’ net energy (kCal) | 373.9±92.9 | 303.2±112 | 374.9±85.4 | 293.2±129 |
| Meals’ net fat (g) | 16.51±4.29 | 13.48±5.03 | 16.58±3.89 | 12.97±5.96 |
| Meals’ net sodium (mg) | 342.3±88.1 | 262.2±122 | 339.8±82.9 | 263.6±140 |
| Meals’ net potassium (mg) | 555.1±141 | 451.2±192 | 562.6±128 | 415.3±206 |
| Meals’ net carbohydrates (g) | 33.27±8.75 | 28.58±7.66 | 33.41±8.10 | 27.66±9.48 |
| Meals’ net protein (g) | 23.66±5.87 | 18.58±7.93 | 23.67±5.72 | 18.09±8.33 |
| *significantly different across SNAQ groups | | | | |

Supplemental Figure 5. Appetite scores across different groups: frailty as assessed by FFS (panel A), robustness as assessed by FFS (panel B), nutritional status as assessed by MNA-SF (panel C), nutritional status as assessed by SNAQ (panel D). Color indicates the frailty group: non-frail (red), pre-frail (green), and frail (blue), or the robustness group: robust (red), non-robust
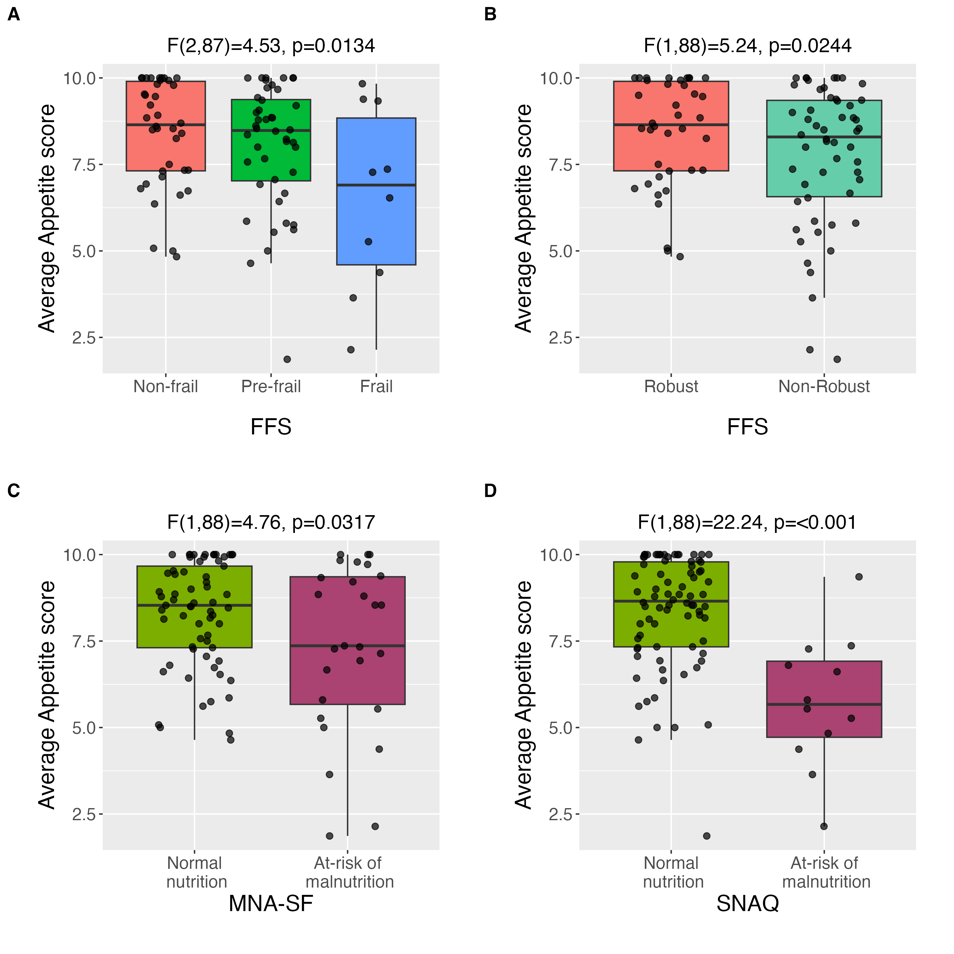
(cyan), or the nutritional group: normal nutrition (green), at-risk of malnutrition (purple). Data are reported in boxplot representation: the central horizontal lines represent the median, the boxes mark the interquartile range, black lines are the whiskers, and the black dots indicate outliers. Title reports F-score, degrees of freedom, and the p-value of the ANOVA test checking the association between average endpoints and groups. The appetite score allows to differentiate across frailty, robustness, and nutritional groups. Frail participants show significantly lower appetite score compared to non-frail (6.51±2.62 vs 8.41±1.56, p=0.0044), and participants at-risk of malnutrition shows significantly lower appetite score, compared to participants with normal nutritional status (6.59±2.40 vs 8.47±1.42, grouped by SNAQ).


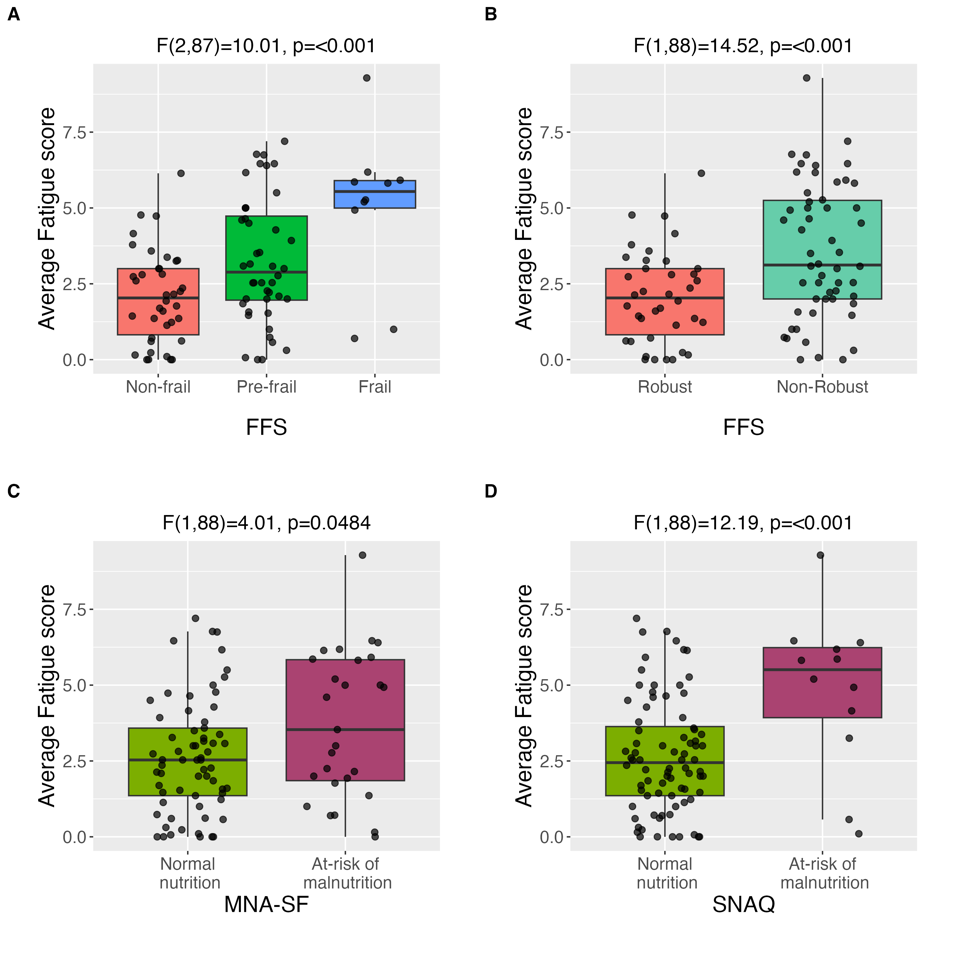


Supplemental Figure 6. Fatigue scores across different groups: frailty as assessed by FFS (panel A), robustness as assessed by FFS (panel B), nutritional status as assessed by MNA-SF (panel C), nutritional status as assessed by SNAQ (panel D). Color indicates the frailty group: non-frail (red), pre-frail (green), and frail (blue), or the robustness group: robust (red), non-robust (cyan), or the nutritional group: normal nutrition (green), at-risk of malnutrition (purple). Data are reported in boxplot representation: the central horizontal lines represent the median, the boxes mark the interquartile range, black lines are the whiskers, and the black dots indicate outliers. Title reports F-score, degrees of freedom, and the p-value of the ANOVA test checking the association between average endpoints and groups. The fatigue score allows to differentiate across frailty, robustness, and nutritional groups. The average fatigue score showes a positive trend towards frailty (non-frail: 2.07±1.53, pre-frail: 3.24±2.05, frail: 5.02±2.51), and the Tukey HSD test shows statistical differences between non-frail vs pre-frail (p<0.01), non-frail vs frail (p<0.001), and pre-frail vs frail (p<0.05). Similarly, participants at-risk of malnutrition shows significantly higher fatigue scores, as compared to participants with normal nutritional status (3.88±2.45 vs 2.66±1.90, grouped by SNAQ).


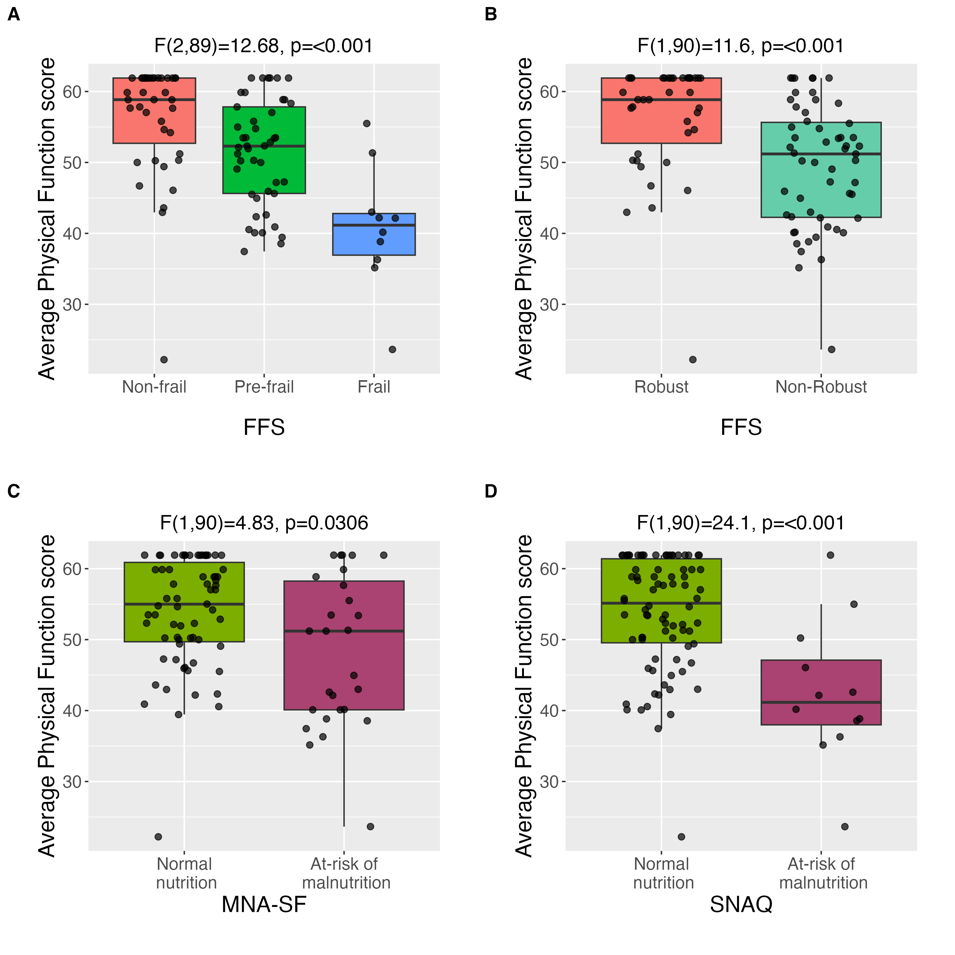
Supplemental Figure 7. PROMIS Physical Function 10a t-scores across different groups: frailty as assessed by FFS (panel A), robustness as assessed by FFS (panel B), nutritional status as assessed by MNA-SF (panel C), nutritional status as assessed by SNAQ (panel D). Color indicates the frailty group: non-frail (red), pre-frail (green), and frail (blue), or the robustness group: robust (red), non-robust (cyan), or the nutritional group: normal nutrition (green), at-risk of malnutrition (purple). Data are reported in boxplot representation: the central horizontal lines represent the median, the boxes mark the interquartile range, black lines are the whiskers, and the black dots indicate outliers. Title reports F-score, degrees of freedom, and the p-value of the ANOVA test checking the association between average endpoints and groups. The average t-score allows to differentiate across frailty, robustness, and nutritional groups. The average t-score shows a negative trend towards frailty (non-frail: 56.26±8.04, pre-frail: 51.36±7.44, frail: 40.83±8.74), and the Tukey HSD test shows statistical differences between non-frail vs pre-frail (p=0.023), non-frail vs frail (p<1e-5), and pre-frail vs frail (p<0.001). Similarly, participants at-risk of malnutrition shows significantly lower physical function t-scores, as compared to participants with normal nutritional status (46.66±11.7 vs 54.21±7.01, grouped by SNAQ).


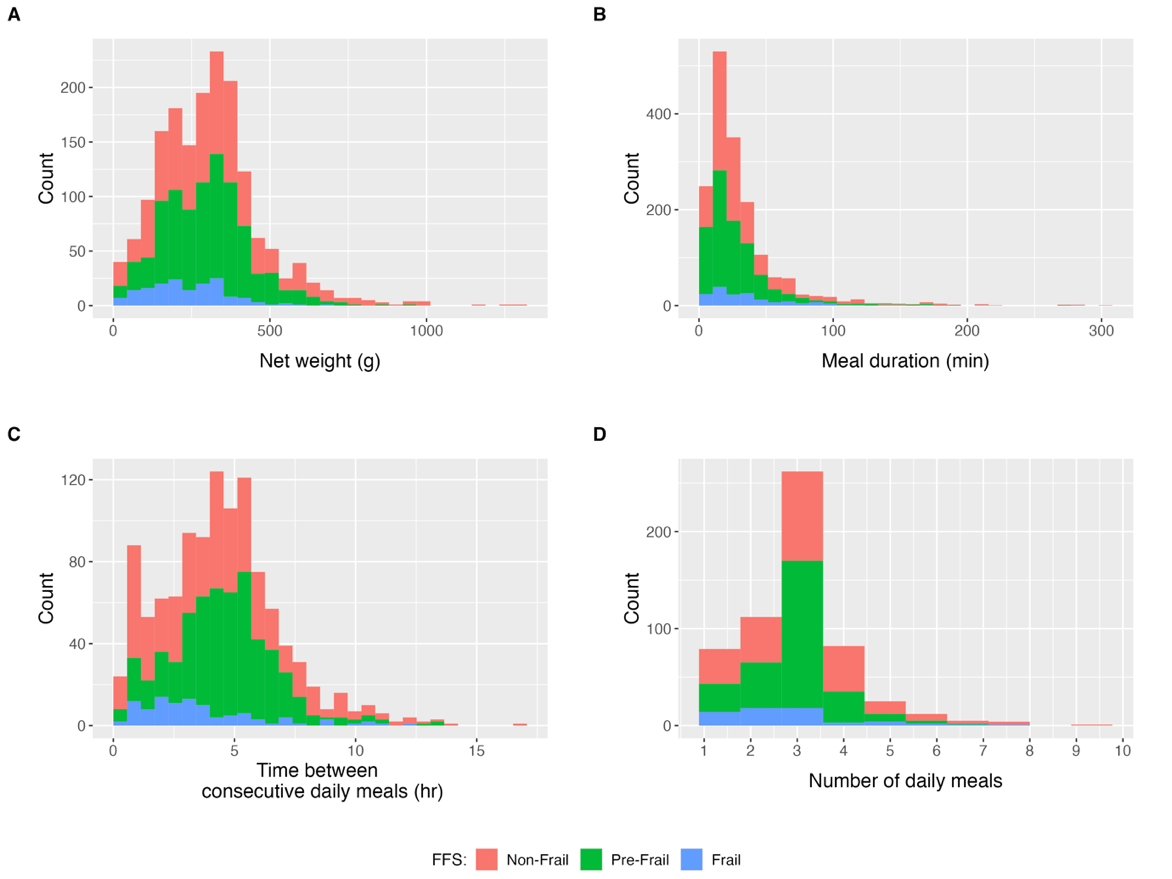


Supplemental Figure 8. Histograms of meals’ net weight (panel A), meal duration (panel B), time between consecutive daily meals (panel C), and number of daily meals (panel D), colored by frailty group (red: non-frail, green: pre-frail, blue: frail).

**Additional information of the Geriatric Anorexia Studies**

The following assessment were performed either in GAS-1 or in GAS-2, as explained in the following:

- Barthel Index was performed in GAS-2 at Visit 1 to measure basic function in activities of daily living, including self-maintenance skills such as dressing, bathing, and grooming.
- Berg Balance Scale was performed in GAS-2 at Visit 1 to assess postural balance.
- Edinburgh Handedness Inventory was performed in GAS-1 and GAS-2 at Visit 1 to assess individuals’ handedness.
- Fried Frailty Assessment was performed in GAS-1 at Visit 1, and in GAS-2 at Visit 1 and at Visit 3 to assess individuals’ frailty status. For GAS-2 participants, the outcome collected at Visit 3 was used for analysis, as participants were supposedly more aware of their frailty status at the end of the monitoring period. Only for one participant whose assessment was not performed at Visit 3, the outcome collected at Visit 1 was used for analysis.
- Geriatric Depression Scale Short Form was performed in GAS-1 at Visit 1 and Visit 2, and in GAS-2 at Visit 1 to measure individuals’ depression state. For GAS-2 participants, the outcome collected at Visit 1 was used for baseline summaries.
- Mini Nutritional Assessment Short Form was performed in GAS-1 at Visit 1, and in GAS-2 at Visit 1 and Visit 3 to assess individuals’ nutritional status. For GAS-2 participants, the minimum score across the two visits was used for analysis, in order to maximize the number of malnourished participants.
- Mobile Device Proficiency Questionnaire-16 was performed in GAS-1 and GAS-2 at Visit 1 to assess device proficiency.
- Montreal Cognitive Assessment was performed in GAS-1 and GAS-2 at Visit 1 to assess cognitive impairment in the following domains: visuospatial, naming, attention, language, abstraction, delayed recall, and orientation.
- Short Physical Performance Battery was performed in GAS-1 at Visit 1, and in GAS-2 at Visit 1 and Visit 3 to evaluate the functional capability and performance of the lower extremity, based on three timed functional components: standing balance, gait speed, and chair stand. For GAS-2 participants, the mean outcome across the two visits was considered.
- Simplified Nutritional Appetite Questionnaire was performed in GAS-1 at Visit 1 and Visit 2, and in GAS-2 at Visit 1 and Visit 3 to assess appetite and risk of weight loss. The minimum score across the two visits was considered for analysis.
- Timed Up and Go was performed in GAS-1 at Visit 1, and in GAS-2 at Visit 1 and Visit 3 to evaluate motor performance based on a sequence of sit-to-stand, walking, turning, and stand-to-sit tasks. For GAS-2 participants, the mean outcome across the two visits was considered.
- WRAT-4 Word Reading was performed in GAS-1 and GAS-2 at Visit 1, to measure reading skills ability.

In addition, a Comfort and wearability questionnaire was deployed in both studies at the last visit, to evaluate participants’ self-reported comfort with the various DHTs. The questionnaire included the following 10 items: 1) Ability to wear/use all devices for the entire duration of the study; 2) Ease of wearing/using each device; 3) Rate the experience using Renpho Body Scale; 4) Rate the experience using Renpho Nutrition Scale; 5) Rate the experience using iPhone app; 6) Example of study activity particularly easy; 7) Rate the experience with provisioned meals; 8) Rate the experience following a regimented meal plan; 9) Example of study activity that would do differently; 10) For each device used in the study, indicate willingness of using it in future studies and for how long.

The differences in inclusion/exclusion criteria between GAS-1 and GAS-2 are summarized as follows: (1) inclusion for GAS-1 age range 65-85, GAS-2 age range 65 and older; (2) inclusion for GAS-1 no recent hospitalizations/acute events in the last 12 months, GAS-2 no recent hospitalizations/acute events in the last 30 days; (3) inclusion for GAS-1 only, no food aversion or food allergies; (4) inclusion for GAS-2 only, MoCA score of ≥ 19; (5) inclusion for GAS-2 only, normal or corrected to normal hearing; (6) exclusion for GAS-2 only, life expectancy of less than 6 months or actively enrolled in a palliative care program; (7) exclusion for GAS-2 only, modified Barthel Index score of < 40.

In GAS-1, the GENEActiv wrist accelerometer was positioned on the nondominant wrist, while in GAS-2 two devices were used and positioned on both wrists, although only data from the nondominant wrist was used to compute physical activity measurements.

Meals whose meal duration was shorter than 1 min or longer than 300 min were considered users’ error; these meals were kept for analysis, but the post-meal time was edited to reflect a meal duration equal to the median meal duration across meals per participant. In addition, meals with pre-meal time recorded 30 min apart were assumed to be fragments of the same meal event (e.g., main course and dessert) and incorporated together, as already performed in the literature. The resulting meal was assumed to have the pre-meal time of the first meal consumed, the post-meal time of the last meal consumed, and net weight and macronutrient content of the fragmented meals were summed up. Finally, meals with resulting net weight lower than or equal to 0 g were considered users’ or devices’ error and excluded from analysis.

In this data, two meals had negative net weight value, resulting in 1690 meals recorded via Renpho Nutrition Scale or iPhone, consumed by 50 participants across a total of 643 monitoring days available for analysis.

## References

1. Powell-Wiley TM, Poirier P, Burke LE, et al. Obesity and cardiovascular disease: a scientific statement from the American Heart Association. Circulation 2021;**143**(21):e984-e1010 doi: https://doi.org/10.1161/CIR.0000000000000973.
2. Johnson Stoklossa CA, Forhan M, Padwal RS, Gonzalez MC, Prado CM. Practical considerations for body composition assessment of adults with class II/III obesity using bioelectrical impedance analysis or dual-energy X-ray absorptiometry. Current obesity reports 2016;**5**:389-96 doi: https://doi.org/10.1007/s13679-016-0228-5.
